# Comparative Genomics and Physiological Investigation of a New *Arthrospira/Limnospira* Strain O9.13F Isolated from an Alkaline, Winter Freezing, Siberian Lake

**DOI:** 10.3390/cells10123411

**Published:** 2021-12-03

**Authors:** Agnieszka E. Misztak, Malgorzata Waleron, Magda Furmaniak, Michal M. Waleron, Olga Bazhenova, Maurycy Daroch, Krzysztof F. Waleron

**Affiliations:** 1 Laboratory of Plant Protection and Biotechnology, Intercollegiate Faculty of Biotechnology UG and MUG, University of Gdansk, 80-307 Gdansk, Poland; amisztak@uliege.be; 2 Department of Pharmaceutical Microbiology, Faculty of Pharmacy, Medical University of Gdansk, 80-416 Gdansk, Poland; magda.furmaniak@acteryon.com (M.F.); michal.waleron@gumed.edu.pl (M.M.W.); 3 Department of Ecology, Nature Management and Biology, Omsk State Agrarian University Named after P.A. Stolypin, 644008 Omsk, Russia; olga52@bk.ru; 4 School of Environment and Energy, Peking University Shenzhen Graduate School, Shenzhen 518055, China; m.daroch@pkusz.edu.cn

**Keywords:** *Arthrospira*, haloalkalotolerant cyanobacteria, metagenomics, phylogenomics, fatty acid

## Abstract

Cyanobacteria from the genus *Arthrospira/Limnospira* are considered haloalkalotolerant organisms with optimal growth temperatures around 35 °C. They are most abundant in soda lakes in tropical and subtropical regions. Here, we report the comprehensive genome-based characterisation and physiological investigation of the new strain O9.13F that was isolated in a temperate climate zone from the winter freezing Solenoye Lake in Western Siberia. Based on genomic analyses, the Siberian strain belongs to the *Arthrospira/Limnospira* genus. The described strain O9.13F showed the highest relative growth index upon cultivation at 20 °C, lower than the temperature 35 °C reported as optimal for the *Arthrospira/Limnospira* strains. We assessed the composition of fatty acids, proteins and photosynthetic pigments in the biomass of strain O9.13F grown at different temperatures, showing its potential suitability for cultivation in a temperate climate zone. We observed a decrease of gamma-linolenic acid favouring palmitic acid in the case of strain O9.13F compared to tropical strains. Comparative genomics showed no unique genes had been found for the Siberian strain related to its tolerance to low temperatures. In addition, this strain does not possess a different set of genes associated with the salinity stress response from those typically found in tropical strains. We confirmed the absence of plasmids and functional prophage sequences. The genome consists of a 4.94 Mbp with a GC% of 44.47% and 5355 encoded proteins. The *Arthrospira/Limnospira* strain O9.13F presented in this work is the first representative of a new clade III based on the 16S rRNA gene, for which a genomic sequence is available in public databases (PKGD00000000).

## 1. Introduction

*Arthrospira* Stizenberger ex Gomont 1892 [1]/*Limnospira* sensu Nowicka-Krawczyk [2] that was formerly called *Spirulina* [3] is a non-nitrogen fixing, filamentous cyanobacterium representing edible, haloalkalotolerant organisms. The genus *Arthrospira* has been observed to occur worldwide in a varied range of haloalkaline habitats, from freshwater alkaline conditions to hypersaline environments [1,4,5]. In tropical and subtropical regions, it forms exceptionally high blooms turning soda lakes into one of the world’s most productive ecosystems [6]. The occurrence of *Arthrospira* has been reported all around the globe (apart from polar regions) from the natural water bodies characterised by a high average temperature during summer and not entirely freezing during winter. Such environments are found throughout the world in Africa’s Rift Valley, in Central Asia, in the North American Desert, on the Andean Plateau, in the rain-shadowed regions of California and Nevada, on the Central Mexican Plateau, on a high-altitude wetland in the Chilean Altiplano, in Manitoba (Canada), in Wadi al Natrun (Egypt), on the Decan Plateau (India), as well as in salty puddles (in Baranda, Europe), in a tropical crater lake located on Mayotte Island (Comoros Archipelago, Western Indian Ocean), in Western Siberia (Russia) and in Eastern Australia [5,6,7,8,9,10,11]. In contrast, the commercial biomass of *Arthrospira* is cultivated only in areas with high yearly average temperatures due to economic reasons. The comparative metagenomic analyses of water, sediments and mats from Canadian, Siberian and American soda lakes revealed a common microbiome characteristic for this haloalkaline environment [8,10,12,13]. Interestingly, the genus *Arthrospira/Limnospira* is dominant in tropical soda lakes in Africa. In contrast, it is absent in the Southern Siberian soda lakes (Kulunda Steppe, Altai) [14] and has not been observed in this area since 1931 when N.N. Voronihin described *Spirulina/Athrospira fusiformis*. However, *Arthrospira* was detected in Solonoye Lake in Western Siberia [7].

The nutritional value of the *Arthrospira* biomass is commonly known and well documented. Besides its nutritional properties, *Arthrospira* evinces therapeutic and industrial potential. Biological activity of several *Arthrospira* compounds, namely, C-phycocyanin, γ-linolenic acid (GLA), calcium spirulan and immulina have all been documented in the fields of cancer-fighting, immunomodulation, cardiovascular diseases prevention and others [15]. In the era of alternative energy resources, the *Arthrospira* biomass is also a promising biofuel, producing mostly hydrogen [16,17,18,19]. *Arthrospira* sp. PCC 8005 is also a part of the fourth compartment of the Micro-Ecological Life Support System Alternative (MELiSSA), aiming to elaborate artificial ecosystems for space expeditions [20,21]. The biomass of *Arthrospira* is commercially available under the name ‘Spirulina’ due to a taxonomic revision proposed by Geitler in 1932 [3] that was later shown to be wrong [22]. Since then, it has been shown that many strains listed as *Spirulina* belong to *Arthrospira* [23]. However, this commercial name is not likely to be replaced by the correct one in the foreseeable future due to its practical and technological meaning [24]. The taxonomy of *Arthrospira* was primarily based on morphology, although ecology and the geographic origin of the organisms was also partly considered to distinguish many species [23,25]. However, the distinction of the *Arthrospira* species based on morphology is questionable and can be misleading, since, e.g., the helix geometry and gas vacuole presence can be influenced by physical and chemical conditions [26,27,28].

So far, for the genetic characterisation and taxonomy of *Arthrospira* only three genetic markers: the 16S rRNA gene, 16S-23S rRNA Internally Transcribed Spacer (ITS) and phycocyanin operon fragment *cpcBA* have been used. The phylogenetic studies of *Arthrospira* based on the 16S rRNA gene divided the genus into three clads (Comte et al. 2013). Meanwhile, only two clusters (I and II) and five subclusters (I.A, I.B, II.A, II.B, II.A/II.B) have been discriminated based on the sequence polymorphism of the ITS [29,30]. The intrageneric relatedness has also been assessed by a sequence analysis of a region of the phycocyanin operon, consisting of the intergenic spacer (IGS) and parts of the flanking *cpcB* and *cpcA* genes [5,31,32,33]. According to Papapanagiotou and Gkelis [34], the combined analysis of molecular (16S rRNA, ITS and *cpcB*A-IGS sequences) and phenotypic (13 morphological and morphometric) characters proposed and described by [35] data were consistent and divided the *Arthrospira* strains into three clusters/taxa. Unfortunately, it is difficult to assess whether the genetic relationship within the genus *Arthrospira* is the same for all three markers, as the phylogenies based on each of them were carried out on different sets of strains. It should be emphasised that the 16S rRNA analysis [2] was not supported by other markers that are typically used in the phylogenetic analysis of cyanobacteria, such as the ITS or phycocyanin operon fragment *cpcBA*. Moreover, since there are no isolates of *Arthrospira jenneri* strains observed in an environmental sample by [2] or deposited in culture collections, further analyses of the biochemical properties and genetic traits that would allow the comparison of both the *Athrospira/Limnospira* genera are not possible. Likewise, no phylogenomic analyses can be performed. Due to the lack of verified isolated strains and/or original genetic material of *A. jenneri*, which can be used for comparative analysis, it is debatable whether this reclassification is accurate. The modern taxonomy of prokaryotes that can be cultivated in vitro should not be based solely on the study of only one or a few genes nor exclusively based on their morphological features, which, in the case of *Arthrospira*, are highly variable depending on numerous factors, which have been accurately documented in the literature [36]. Therefore, in this work, in which we use genomic data for the classification of a new Siberian isolate, we retain the taxonomic name *Arthrospira*.

Up to now (7 October 2021), 17 *Arthrospira* genomes have been sequenced: *Arthrospira* sp. PCC 8005 [37], *A. platensis* NIES-39 [38], *A. platensis* C1 [39], *A. maxima* CS-328 [40], *A. platensis* Paraca P0 [41], *A. platensis* YZ [42], *Arthrospira* sp. TJSD091 [43], *Arthrospira* sp. PLM2.Bin9 [13,44], *A. platensis* NIES-46 [19], *A. platensis* FACHB-835 [45], *A. fusiformis* SAG 85.79 [31], *Arthrospira* sp. BM01 [46], *A. fusiformis* KN (JACGXW000000000), *A. platensis* FACHB-971 [45], *A. platensis* FACHB-439 [45], *Arthrospira* sp. PCC 9108 (CP066886), *Arthrospira* sp. SH-MAG29 [9] and *Arthrospira* sp. TJSD092. This publicly available genomic data can be used for comparative genomics and phylogenomic analyses allowing a reliable identification of the newly isolated Siberian strain O9.13F.

This study aimed to perform a comprehensive genetic and physiological investigation of the new *Arthrospira/Limnospira* sp. strain O9.13F isolated from an alkaline, winter freezing Siberian lake. The study aimed (1) to classify and determine the systematic position of the new strain, (2) to characterise physiologically the strain capable of surviving the winter period in the Siberian lake, (3) to perform a comparative genomic analysis of Siberian and tropical *Arthrospira/Limnospira* strains and (4) to verify whether the O9.13F strain is suitable for cultivation in the temperate climate zone.

## 2. Materials and Methods

### 2.1. Strain Isolation and Growth Conditions

On 11 September 2013, the thick cyanobacteria bloom from Solenoye Lake (Supplementary Figure S1) was collected into sterile, capped containers and transported to the laboratory. Then the biomass was resuspended in a liquid Zarrouk medium [47] and treated with trimethoprim (60 µg/mL), cycloheximide (200 µg/mL) and amphotericin B (5 µg/mL) to remove fungi. Next, a series of repassages on the solid Zarrouk medium was performed to separate cyanobacteria present in the bloom from each other and co-occurring bacteria. The axenity of the *Arthrospira* cultures was examined according to the procedure recommended by Rippka [48].

Moreover, to be certain that the new strains were unicyanobacterial, eight single filaments with different morphologies were isolated from the fresh biomass from an alkaline pond. From each of the filaments, pure cyanobacterial cultures (named O9.13A-H) were obtained. All new isolates were identified by sequencing of the 16S rRNA gene and screened by PCR with primers specific for the ITS subclusters [29,30] and the ITS_CL_III primer designed in this work. As the newly isolated strains were morphologically and genetically identical, only the O9.13 F strain was selected for further analyses and remained a part of the *Arthrospira* strains collection at the Intercollegiate Faculty of Biotechnology, University of Gdansk, Poland.

To determine the optimal growing conditions, the *Arthrospira* culture was grown with and without shaking at 150 rpm in the Zarrouk liquid medium pH~10–11 under a 16 h light/8 h dark photoperiod under illumination of 30–40 µmol photons m^−2^ s^−1^ provided by fluorescent lamps at four temperatures: 15 °C, 20 °C, 28 °C and 35 °C and in three salinities, NaCl concentration of 25 g/L, 50 g/L and 100 g/L, in triplicates, starting with the culture of similar optical density (3 McF in the case of strain O9.13F). Two other *Arthrospira* reference strains, PCC 8005 and PCC 7345, were grown in the same conditions, except temperature 15 °C at which these strains die off. The growth index was expressed as a ratio of the initial culture optical density to the final measured value of a particular sample after 8 days of incubation (averaged from all replicates). To assess the properties of the biomass, the fatty acids (FA), biosynthetic pigments and proteins were extracted from the above-mentioned cultures.

### 2.2. Phenotypic Characteristics of Arthrospira sp. O9.13F

#### 2.2.1. Acclimation of *Arthrospira* Strains to Long-Term Stress of Low Temperature

*Arthrospira*, O9.13F and four strains PCC 8005, PCC 7345, SAG 49.88 and CCALA 023 were cultivated without shaking under cold room conditions in the Zarrouk medium under a constant lighting of 10 µmol photons m^−2^ s^−1^ with a daily amplitude of 5–8 °C for 10 months. After 3 and 10 months of cultivation in the cold room, all samples were inoculated onto the solid Zarrouk medium and grown at 20 °C under a light intensity of 20 µmol photons m^−2^ s^−1^ for two months. The photographic documentation was made using a Nikon 80i light microscope.

#### 2.2.2. The Survivability of *Arthrospira* Strains in Different Stress Conditions

The *Arthrospira* strains O9.13F, PCC 8005 and PCC 7345 were subjected to stress conditions caused by the addition of different substances, cultivation in water acquired from industrial ponds or changes in medium salinity. The stress factors were selected based on several criteria: (i) They can appear in the natural pond next to the farmlands. (ii) The used stressor would be useful for the removal of an excess of associated microorganisms from the biomass before various procedures (e.g., genome sequencing). (iii) They would be beneficial in growing in numerous industrial and natural ponds. The tested stressors and conditions included: working solution of fungicide 0.35% (Curzate Cu 49.5 containing cymoxanil and copper oxychloride), working solution of herbicide 0.75 mg/L (glyphosate), working solution of insecticide 0.04% (Clothianidin), hydrogen peroxide 50, 100 and 200 mM, silver nanoparticles (AgNPs) 3 μg/mL, industrial water rich in calcium, potassium and sodium carbonate with pH = 13 (Poland); undiluted and industrial water mixed 1:1 with a Zarrouk medium, water from Gdańsk Bay (estimated 7–8‰ of salinity) and the addition of NaCl to total salinity 150 and 200 g/L. The biomass was cultivated at 28 °C under stress for one week. Afterwards, the biomass was washed with distilled water and transferred to a solid Zarrouk medium. Due to the filamentous character of the *Arthrospira* biomass, as well as the tendency of the biomass to create mats, a standard means of measuring bacterial growth (optical density) is not applicable for assessing the growth kinetics of this organism. Therefore, a method based on the condition, duration of the lag phase and visible increase in the number of viable filaments was developed. Here, the recovery rate was defined as to whether the trichomes survived the stress conditions and, if so, after what time the active growth was resumed upon reintroduction to physiological conditions. This process was monitored using the following criteria: 0—degradation of the filaments, 1—whole, nonfragmented filaments present, no growth observed, 2—some growth observed after two weeks of a lag phase, 3—weak growth after one week of a lag phase, 4—stable growth after a short lag phase and 5—abundant growth without a lag phase.

#### 2.2.3. Fatty Acids Methyl Ester Analysis (FAME)

The total lipids were extracted in triplicates according to the Bligh and Dyer protocol [49] by homogenising the biomass in chloroform/methanol under ultrasonication. Following evaporation, the lipids were derivatised to FAMEs with sulphuric acid (2%) in methanol. The resulting FAMEs were extracted to hexane and analysed using a Shimadzu GC-2010 equipped with a flame ionization detector and a 60 m × 0.25 mm BPX70 column (SGE Analytical Science). Standard mixtures of fatty acids methyl esters (Supelco^®^ 37 Component FAME Mix, 1890, cis/trans) were used to identify the major fatty acids based on their retention time. Individual FAs were expressed as percentages of the total FAs present in the chromatogram.

#### 2.2.4. Proteins Content Analysis

The total amounts of proteins in the extracts obtained by ultrasonication of the biomass were measured following the protocol of Bradford [50]. The 1 mL samples (triplicates) of the culture of *Arthrospira* sp. O9.13F cultivated in various conditions were carefully washed with distilled water three times and centrifuged for 20 min at 13,200× *g* rpm. The cell lysis was performed with 1 mL of 0.1 M NaOH, and the cells were incubated for 30 min at 95 °C with shaking. Afterwards, the tubes were left to cool down, acidified with a 5 M solution of HCl and centrifuged for 10 min under 13,200 rpm. To each well in the 96-well plate, 250 μL of the Bradford reagent (Sigma Aldrich #B6916) and 10 μL of the samples (in triplicates) were added. The spectrophotometric measurements were done on an Envision 2105 Multimode Plate Reader (Perkin Elmer) after 30 min of incubation at room temperature without shaking in the wavelength 595 nm. The protein concentration was determined by comparison to a standard curve prepared based on a BSA solution in the range between 0.1–1.4 mg/mL.

#### 2.2.5. The Measurements of Photosynthetic Dyes Content

The biomass obtained from 1 mL of the tested culture in triplicates was centrifuged for 20 min at 13,200× *g* rpm, the supernatant was removed, and 1 mL of 90% methanol was added. The biomass was further lysed by ultrasonication for 20 min and centrifuged again for 15 min at 13,200× *g* rpm. The absorbance was measured by the spectrophotometer at 470 nm, 665 nm and 720nm. The concentration of chlorophyll *a* and total carotenoids was established according to equations by [51,52]:

Chlα [μg/mL] = 12.9447 × (A_665_ − A_720_)

Carotenoids [µg/mL] = 1000 × (A_470_ − A_720_) − 2.86 Chlα [µg/mL]/221

#### 2.2.6. Statistical Analysis

The analysis of differences between the groups was performed using the Shapiro–Wilks test followed by Kruskal–Wallis test using the criterium of Fisher’s least significant difference (LSD) in R [53].

### 2.3. Microscopic Observations

#### 2.3.1. Light Microscopy

The microscopic observations of *Arthrospira* sp. O9.13F filaments were performed, and the photographs were taken with a Nikon 80i microscope with a Nomarski contrast at ×400 and ×1000 magnification with the NIS Elements version D software from Nikon.

#### 2.3.2. Transmission Electron Microscopy (TEM)

Preparation for the ultrathin sectioning and preparation for TEM were carried out as described previously [23,54,55]. Samples were fixed in 2.5% formaldehyde and 2.5% glutaraldehyde in a 0.05 M cacodylate buffer (pH 7.0) for 4 h at room temperature. Then, the material was rinsed in the same buffer and postfixed in 1% osmium tetroxide in the cacodylate buffer at 4 °C overnight. Specimens were treated with 1% uranyl acetate in distilled water for 1 h, dehydrated in an acetone series and embedded in Spurr’s resin. Serial ultrathin (60–100 nm) sections were cut with a diamond knife on a Sorvall MT2B microtome. The material on the grids was poststained with a saturated solution of uranyl acetate in 50% ethanol and 0.04% lead citrate. Observations were made using a Philips CM 100 transmission electron microscope operating at 80 kV. For the scanning electron microscope, samples from fresh cultures were fixed in 2.5% formaldehyde and 2.5% glutaraldehyde in 0.05 M Na-cacodylate buffer (pH = 7.0) for 4 h at room temperature, dehydrated in an ethanol series (30, 50, 75, 96, 100%) and critical point dried using liquid CO_2_ and a K850 critical point dryer (EMITECH, Ashford, England). The dried specimens were mounted on stubs with SPI carbon conductive double-sided adhesive discs, gold coated (350 Å) (SPI-Module Sputter Coater, Structure Probe, Inc., Chester, PA, USA) and examined in a Phillips XL-30 SEM operating at an accelerating voltage of 15 kV.

### 2.4. Molecular Characteristics

#### 2.4.1. DNA Extraction

For DNA extraction, 10 mL of the dense *Arthrospira* culture cultivated at 20 °C was centrifuged, and the collected biomass was carefully washed 3 times with sterile distilled water prior to nucleic acid extraction. Afterwards, the cell lysis and nucleic acids extraction were carried out according to the protocol proposed by the Joint Genome Institute for bacterial DNA isolation using CTAB [56] followed by RNA digestion using Turbo RNase (Ambion). The DNA quantity and quality were assessed first using a NanoDrop Spectrophotometer and later with agarose gel electrophoresis.

#### 2.4.2. PCR Amplification Sequencing and Phylogenetic Analysis

The sequences of the *cpcBA*, ITS and 16S rRNA gene of the new Siberian isolates were determined after cloning as previously described [57]. To confirm this assignment, one clone for each ribosomal operon variant was sequenced with primers 359F, 16S979F and 23S30R [58,59,60]. For amplification and sequencing of phycocyanin operon, fragment primers described by [32] were used. Sequencing was carried out using an ABI PRISM DNA Sequencer (Perkin-Elmer) according to the manufacturer’s manual. Both strands were sequenced using the forward and reverse PCR primers. Moreover, the clones were assigned to the different ITS clusters as described by [30] with an additional reverse primer ITS_CL_III (TTGACTATCAGAGGAAATCCG) designed by us specific for cluster III based on the sequence from *Arthrospira* PCC9901 (MF509176) and which was used with the primer 16S3′F [29].

Further, the obtained sequences were subjected to a BLAST sequence similarity search analysis to identify the nearest taxa. The obtained sequences of the 16S rRNA gene, *cpcBA* operon, ITS and sequences of the closest representative taxa that belong to cyanobacteria were aligned using the MAFFT algorithm in Geneious v9.1.8. [61]. The phylogenetic analysis was performed with the MEGA v. X software, (www.megasoftware.net (accessed on 23 October 2020)), and trees were constructed using the Neighbour Joining algorithm with a Tamura 3-parameter and Maximum Likelihood algorithm, and the Hasegawa–Kishino–Yano models selected on the model test module were implemented in MEGA. A bootstrap analysis with 1000 replications was performed to assess the robustness of the clusters.

The nucleotide sequence data reported in this paper are available under the following accession numbers: 16S rDNA, MF509165, ITS; MF509166–MF509169, *cpcBA* and MF509119. As the 16S rRNA gene sequences obtained for all eight clones were identical, only one was deposited in the GenBank database. A similar situation occurred with the *cpcBA* operon sequence. In the case of the ITS, the sequences of two different genetic variants obtained for the two clones O9.13F and O913H were submitted.

#### 2.4.3. Whole-Genome Sequencing and Annotation

Genome sequencing was performed on an Illumina HiSeq 2000 platform in the Institute of Biochemistry and Biophysics of the Polish Academy of Sciences, Warsaw, Poland. The genome assembly was performed using computational resources provided by the grid computer “durandal” operated by InBioS-PhytoSYSTEMS at the University of Liège (Belgium).

The raw reads were first filtered using Kraken v1.0 software [62] followed by quality and adapter trimming performed using Trimmomatic v0.32 [63]. The Kmer length and distribution were analysed using kmergenie v1.6949 [64]. Processed reads were de novo assembled using Spades v3.10.1 [65], Velvet v1.2.10 [66], MIRA v1.1.1 [67], Geneious v9.1.8 [61] and Ray v2.3.1 [68]. The resulting sets of contigs were integrated, and the final assembly was corrected by CISA v1.3 (Lin and Liao, 2013) software and finally scaffolded with SSPACE v3.0 [69]. The assembly was evaluated using Quast v4.5 software [70].

Annotation was performed by the NCBI Prokaryotic Genome Annotation Pipeline [71,72]. Functional annotations were carried out by the KEGG Automatic Annotation Server [73] and AutoFACT [74], for which the annotation was based on the COG, Smart and Pfam databases. Signal peptides and transmembrane helices were predicted with a Phobius server [75].

R-M systems were identified using the REBASE^®^ database [76], Clustered Regularly Interspaced Short Palindromic Repeats (CRISPR) sequences were predicted using the CRISPRFinder tool [77]. CRISPR-associated genes used in comparative genomics were obtained from the MaGe platform [78]. The identification of prophage regions was performed using the PHASTER tool [79]. Plasmid sequences were detected using the PlasmidFinder server [80].

The screening for the secondary metabolite biosynthesis gene clusters in the *Arthrospira* sp. strain O9.13F genome sequence was assessed with AntiSMASH [81,82].

#### 2.4.4. Phylogenomic Analyses

The phylogenomic analysis is based on the sequences of 363 of the most conserved proteins comparison using the PhyloPhlAn computational pipeline (v3) [83]. The calculation of the average nucleotide identity (ANI) was performed using the PyAni module (v0.2.10) [84] with MUMmer (v3.23) [85] as an alignment method. In silico DNA–DNA hybridisation (*is*DDH) values were calculated using the Genome-to-Genome Distance Calculator (v2.1) [86] with BLAST+ [87] as a local alignment tool.

## 3. Results

### 3.1. Phenotypic Characteristics of Arthrospira sp. O9.13F

#### 3.1.1. Morphology of *Arthrospira* sp. O9.13F

**Figure 1:**
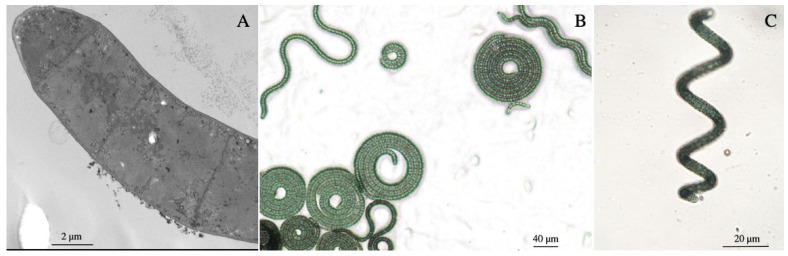
Images of *Arthrospira* sp. O9.13F. Photographs were taken by TEM (**A**) and light microscopy of *Arthrospira* culture grown on solid (**B**) and liquid Zarrouk medium (**C**).

**Figure 2:**
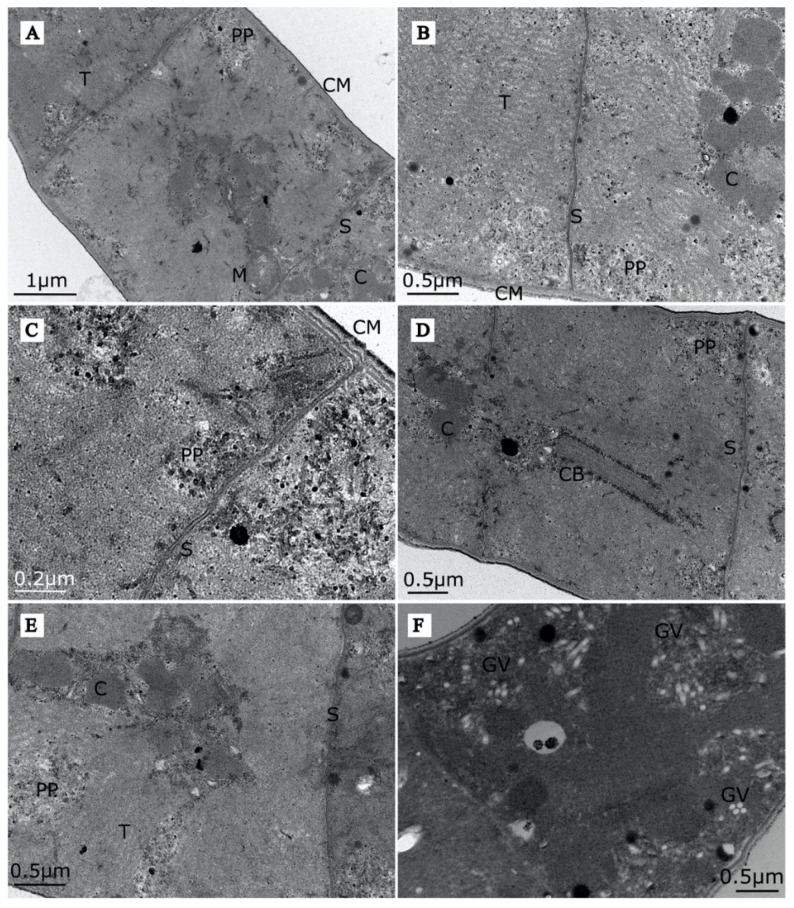
TEM photography of cellular ultrastructures of *Arthrospira* sp. O9.13F. Panels (**A**–**F**) represent different cross-sections of cells illustrating ultrastructures recognised. Abbreviations as follows: cell membrane (CM), septum (S), gas vesicles (GV), thylakoids with bead-like ribosomes (T), mesosome (M), carboxysomes (C), cylindrical bodies (CB), polyphosphate granule (PP).

Upon introduction into the laboratory conditions, trichomes of *Arthrospira* sp. O9.13F were in the shape of tight left-handed open helices (Figure 1). With time and without the influence of natural UV light, the trichomes became looser. Macroscopically, the biomass of strain O9.13F either created a layer just under the surface of the medium or remained on the bottom of the bottle with a tendency to create mats. The trichomes were motile and covered with a layer consisting of exopolysaccharides. The morphology and ultrastructures of the *Arthrospira* sp. O9.13F were typical for representatives of this genus. The TEM (Figure 1A and Figure 2) and light microscopy show the cell dimensions and morphology of the filaments grown on a solid (Figure 1B) and liquid medium (Figure 1C).

We identified all the ultrastructures recognised in other strains of *Arthrospira* investigated by Van Eykelenburg [54] and Tomaselli [23]—thylakoids, cylindrical bodies, mesosomes, gas vesicles, carboxysomes, cyanophycin granules, polyhydroxyalkanoate inclusions, cell membranes and septa (Figure 2A–E). Abundant gas vesicles with a diameter of 45–60 nm and a length up to 600 nm scattered throughout the cells were visible in the EM pictures of strain O9.13F (Figure 2F). The density is comparable to other strains as visible in the TEM microscopic images by [23,54].

#### 3.1.2. Growth Conditions

**Figure 3:**
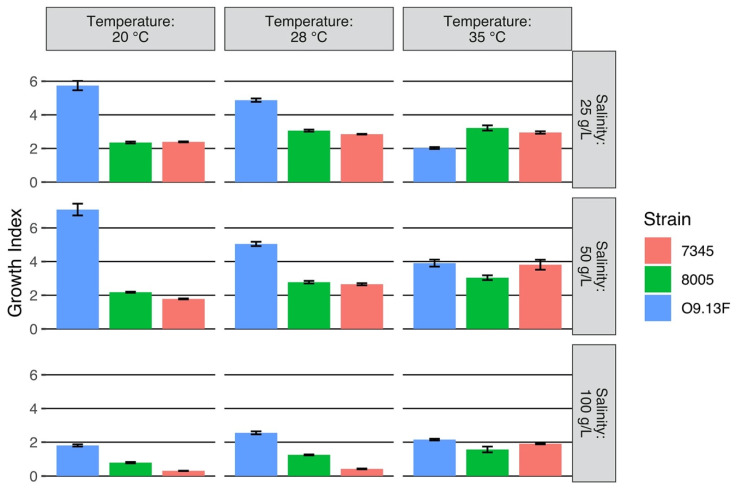
Effect of salinity (Zarrouk medium supplemented with NaCl to total salinity up to 25 g/L, 50 g/L, 100 g/L) on the growth of *Arthrospira* sp. strains, O9.13F, PCC7345 and PCC8005. Strains were grown for 8 days at different temperatures (20 °C, 28 °C and 35 °C). The optical density measurements were performed in triplicates for each strain in each of the conditions. The standard deviations are shown. The growth index is the quotient of the optical density [McF] of the culture after 8 days incubation and the optical density of the culture at the start point of the experiment.

Cell growth occurred at pH 7.0–10.0, and it tolerated from 1 up to 150 (g/L) of NaCl. The growth temperature of the strain O9.13F ranged between 15 and 35 °C, with optimum growth at 20 °C (Supplementary Figure S2). The optimal growth index for O9.13F of 20 °C was lower than the optimal temperature of the tropical strains *Arthrospira* sp. PCC8005 (India) [30] and *A. platensis* PCC7345 (saline marsh Del Mar Slough, CA, USA) for which the largest increase of biomass production was observed at 28 °C (Figure 3). Two reference strains, PCC8005 and PCC7345, were not grown at a temperature of 15 °C because in this condition, both strains stopped their metabolism, and filaments started to fall apart; therefore, the measurements of proteins and fatty acids were below the sensitivity limit of the method. Interestingly, for the strain O9.13F grown at 15 °C, its growth index was higher in the medium with higher salinity. It was around 4 McF in the medium supplemented with 150–200 g/L of NaCl, while at lower concentrations of 25–50 g/L, it reached a maximum of 2.5. The highest growth was observed in the medium with a salinity of 150 g/L (Supplementary Figure S2).

**Figure 4:**
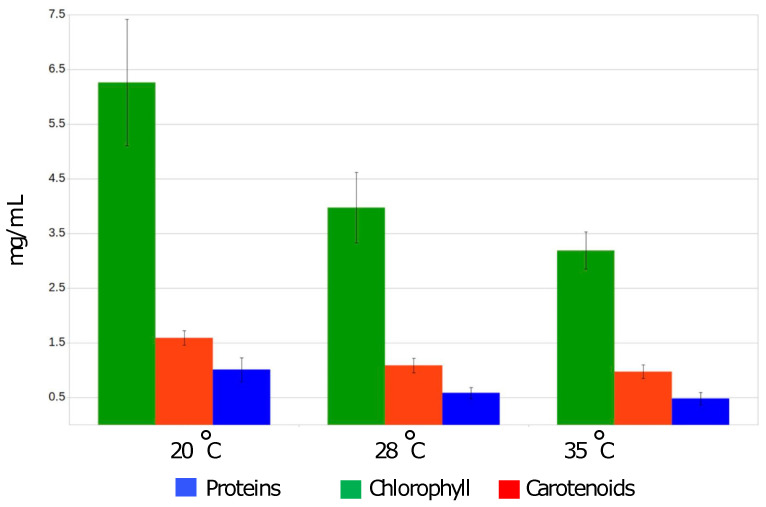
Quantity of chlorophyll, total carotenoids and proteins [mg/mL] extracted from biomass of *Arthrospira* sp. O9.13F cultivated at three different temperatures, 20 °C, 28 °C and 35 °C in Zarrouk medium with a total salinity 25 g/L. Standard deviations based on three replicate analyses are shown.

Accordingly, the highest amount of proteins (1.01 mg ± 0.22 /mL) and photosynthetic dyes: chlorophyll (6.26 ± 1.16 mg/mL) and total carotenoids (1.59 ± 0.13 mg/mL) extracted from the biomass of O9.13F, were obtained from the culture cultivated at 20 °C. Under the conditions of the highest salinity, 100 g/L, all three strains had a significantly lower biomass increase than in other salinity values, regardless of the temperature at which the cultures were grown. However, the biomass grown at 35 °C in the 100 g/L salinity medium was the lowest (Figure 4).

#### 3.1.3. Acclimation to Prolonged Cold Stress

As the strain was isolated from a Siberian lake that is covered by ice in the winter, we tested the tolerance in laboratory conditions at a low temperature and compared it with other strains that originated from warmer regions: PCC8005 (India), PCC7345 (USA), *A. maxima* SAG 49.88 (Italy) and *A. fusiformis* CCALA23 (Kenya). The experiment was performed in a cold room, in low light 10 µmol photons m^−2^ s^−1^ conditions, for 10 months with subculturing after three months.

The general observation was that strains lost the intensity of pigmentation, and some filaments started to decay after this period. One of the strains, *A. maxima* SAG 49.88, changed the morphology and began to coil filaments. After the inoculation of tested strains filaments on a solid Zarrouk medium and transfer to the higher temperature 20 °C with a change of light intensity 20 µmol photons m^−2^ s^−1^, the strains recovered to the normal pigmentation, and growth was visible after a week of incubation; with exception of the strain *A. fusiformis* CCALA 23 that started to grow as a visible mat of filaments on the plate after six weeks (Figure S3).

#### 3.1.4. The Sensitivity of *Arthrospira* sp. O9.13F to Various Stressors

**Figure 5:**
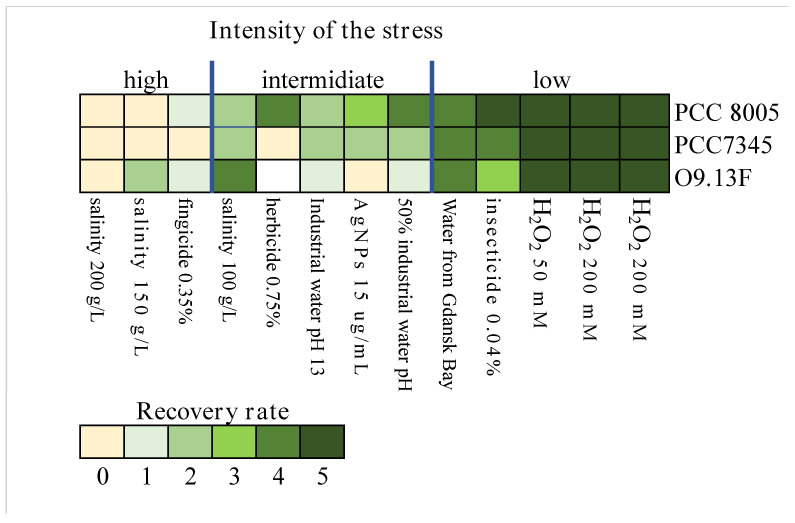
The heatmap presents the recovery rates of eight tested *Arthrospira* strains, O9.13F, PCC 7345 and PCC 8005 cultivated in stress conditions. Above the heatmap, three levels of the stress severity (low, intermediate, high) are grouped according to the overall similarity of the different strains’ responses. The colour scale given in the heatmap key corresponds to the recovery rate. The survivability of the biomass was assessed based on the condition, duration of the lag phase and visible increase in the number of viable filaments and recorded by the following system: 0—degradation of the filaments, 1—whole, nonfragmented filaments present, no growth observed, 2—growth observed after two weeks of a lag phase, 3—very weak growth recorded after one week of a lag phase, 4—stable growth after a short lag phase, 5—very abundant growth without a lag phase.

Based on the survival rate of the *Arthrospira* strains, the tested stress factors were divided into three levels of severity: low, intermediate and high. The best growth (low-stress severity) with no noticeable lag phase after the passage to the fresh medium was observed after the addition of hydrogen peroxide (in all three concentrations). The biomass of all tested strains was also showing minimal inhibition of the growth upon exposition to the working concentration of insecticide, water from Gdańsk Bay (lower salinity) and water from an industrial pond (pH > 13) diluted with a Zarrouk medium 1:1. The most differential and strain-dependent effect (intermediate stress severity) was observed in the case of exposition to undiluted industrial water (reach in calcium, potassium and sodium carbonate and pH > 13), silver nanoparticles (15 µg/mL) and herbicide and salinity of 100 g/L. Among the most harmful factors (high-stress severity) were high salinity, 150 and 200 g/L of total salt and a working concentration of 0.04% of fungicide. From the compilation of the results of the sensitivity of the strains to the applied stresses, we concluded that the most sensitive strain was *Arthrospira* sp. O9.13F (Figure 5).

#### 3.1.5. Fatty Acid Composition

The major fatty acids detected in the *Arthrospira* strains were palmitic acid (16:0), gamma-linolenic acid (18:3n6), linoleic acid (18:2n6c) and palmitoleic acid (16:1). However, the fatty acid content of the *Arthrospira* sp. strain O9.13F was not the same as in the strains PCC8005 and PCC7345. The most significant difference in fatty acid content in the *Arthrospira* biomasses was observed in the percentage of palmitic and gamma-linolenic acid (Table 1). The observed decrease of gamma-linolenic acid in favour of palmitic acid in strain O9.13F could be due to the stressful growth conditions and the fatty acid content. All tree strains were cultivated at 28 °C, which is not optimal for the growth of the Siberian strain O9.13F (Table 1). Indeed, the same observation was recorded when the fatty acid content was established for the *Arthrospira* sp. strain O9.13F grown at 20 °C, 28 °C and 35 °C (Supplementary Figure S4).

### 3.2. Taxonomy of Arthrospira sp. Strain O9.13F

The new Siberian isolate of *Arthrospira* was identified based on the 16S rRNA gene, ITS and the phycocyanin operon fragment *cpcBA* sequencing and three different genomic analyses: ANI, *is*DDH and core proteome-based phylogeny.

The cloning and sequencing of the amplified 16S and *cpcBA* revealed that all eight isolates O9.13A-H (each originated from a single filament) have identical (100%) sequences. As a result of the ITS cloning and sequencing, we obtained two different genetic variants representing the ITS clusters I and III according to the numbering proposed by [30,88], respectively. These observations are in agreement with the results of the detection of the ITS variants by PCR with subcluster-specific primers described by Baurain et al. [30] and the reverse primer ITS_CLIII designed by us.

As the newly isolated strains were morphologically and genetically identical, only strain O9.13 F was used for further analyses. The phylogenetic analyses based on the 16S rRNA gene, ITS and phycocyanin operon fragment *cpcBA* were performed on the same dataset.

The obtained 16S rRNA gene sequence of the isolate O9.13F was 99.91% similar to *A. erdosensis* ‘Inner Mongolia’ (JN831261), *Arthrospira* sp. PCC 9901 (MG777134) and 99.72% similar to the other *Arthrospira* sp. strains TJSD092 (CP028914) and PCC 8005 (FO818640).

The phylogenetic analysis based on the small ribosomal subunit sequence showed that the Siberian strain groups with the strain PCC 9901 belonging to the new III clade described by Comte et al. [88] (Supplementary Figure S5). However, it is worth noting that the sequence 16S rRNA gene of the BM01 strain is only 92.5% similar to the sequences of other *Arthospira* strains, and it was excluded from the analysis.

Likewise, in 16S rRNA-based phylogeny, the analysis of the ITS region showed only two phylogroups within the *Arthrospira* genus called ITS clusters and five different genotypes named subclusters according to Baurain et al. [30]. ITS cluster I gathers strains belonging to clades I and III described based on the 16S rRNA gene, while ITS cluster II consists of all strains from the 16S clade II. Strains belonging to 16S clades I and III formed clearly separated subclusters within ITS cluster I. The results of the cloning and sequencing of ITS from strain O9.13F showed that two different genetic variants (genotypes) had been detected within the chromosome. The ITS sequence of clone I is grouped together with strains from 16S clade I, while the ITS clone III is grouped together with strains from 16S clade III (Supplementary Figure S6).

Based on the sequence of the phycocyanin operon fragment *cpcBA,* the newly described strain O9.13F is grouped with strains that belong to clade I (based on the 16S rRNA gene). However, unlike the results of ITS-based phylogeny, strains carrying the 16S gene sequences belonging to clade III appear to be more closely related to strains with the ITS cluster II.B. Moreover, strains belonging to the ITS subcluster II.A described by Baurain et al. [30] are more related with strains cluster I (Supplementary Figure S7).

**Figure 6:**
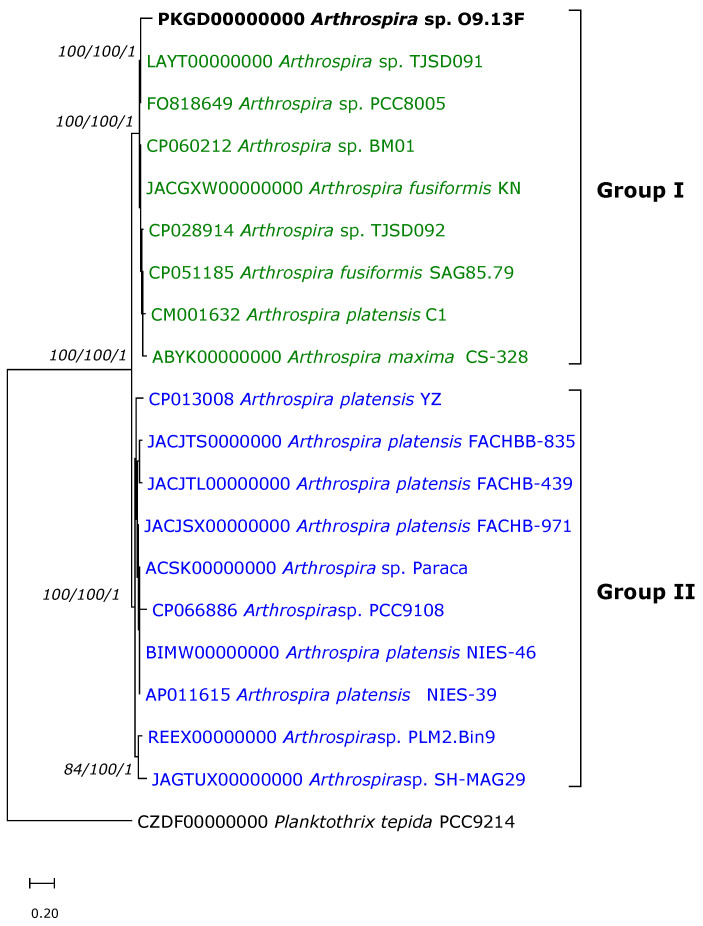
Maximum Likelihood tree indicating the current relatedness of *Arthrospira* sp. O9.13F.

The phylogenomic analysis is based on 363 of the most conserved proteins retrieved from 17 *Arthrospira* strains for which the genomic sequences are available in databases. The use of the most evolutionarily conserved proteins whose genes occur in a single copy in the cell and an analysis based on genomic sequences reduce the impact of intragene or operons recombination on the results of phylogenomic analyses. The topology of the ML tree based on core protein showed that the *Arthrospira* genus consists of only two groups, and the O9.13F strain clusters together with the *Arthrospira* strains PCC8005, TJSD091, TJSD092 and CS-328, all of which are representatives of 16S clade I and ITS cluster I (Figure 6). The presence of two phylogenetic groups revealed by our phylogenomic analyses is consistent with the analyses described above and with the previously described analyses of ITS sequences by Baurain et al. [30]. Phylogenesis based on the analysis of core proteins enabled the precise classification of all strains for which the genomes are known. Also, for strains for which the identification relying on single locus, 16S rRNA gene, was doubtful, e.g., *Arthrospira* sp. BM01. The same goes for strains *A. platensis* Paraca and *Arthrospira* sp. PLM2.Bin9 that were ungrouped when the fragment of the *cpcBA* phycocyanin operon was analysed [31].

The phylogenetic tree establishing *Arthrospira* sp. O9.13F’s phylogenetic position is built based on the sequences of 363 of the most conserved proteins extracted from *Arthrospira* proteomes that are available in the Genbank database. The Maximum Likelihood tree was constructed using the PhyloPhlAn computational pipeline (Segata et al. 2013). The distance tree was inferred using Geneious software v 9.1.8. The gene sequences of *Planktothrix tepida* PCC9214 (CZDF00000000) were used as an outgroup. The bootstrap consensus was inferred from 1000 replicates. Bootstrapping values <50% were cut off. Numbers above branches indicate the bootstrap value for ML, the NJ method and the posterior probabilities for the Bayesian inference method, respectively. The sequence of strain O9.13F is indicated in bold font. The colours correspond to the 16S clades: green -clade 1, blue—clade II (according to [30]) and red—clade III (according to [88]).

**Figure 7:**
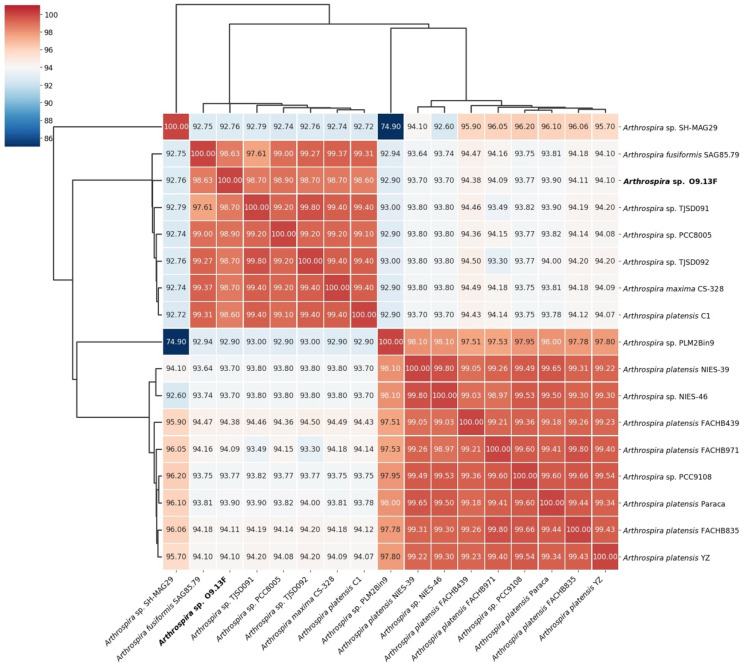
Average Nucleotide Identity (ANI) of 17 *Arthrospira* genomes available in the GenBank database as calculated using PyAni v0.2.10 [84], with MUMer v3.23 [85] as the alignment method. Depicted linkage is calculated as UPGMA, distance is estimated using the Euclidean method.

**Figure 8:**
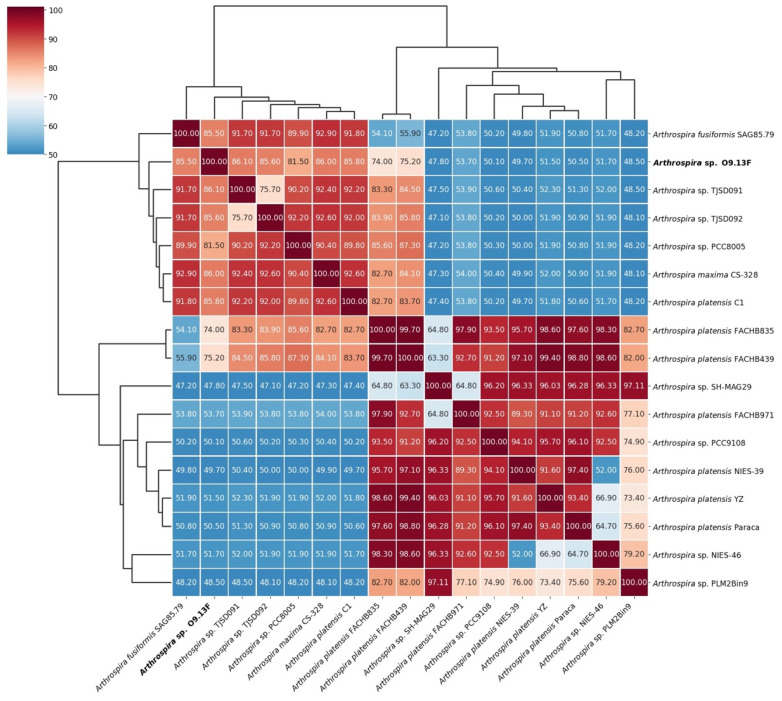
In silico DNA–DNA hybridization of 17 *Arthrospira* genomes available in the GenBank calculated with GGDC 2.1 [86] with BLAST+ as a local alignment tool.

To compare the relatedness of the *Arthrospira* sp. O9.13F to other available genomes from the *Arthrospira* genus, we analysed the Average Nucleotide Identity (Figure 7) and in silico DNA–DNA hybridisation (Figure 8). Both methods confirmed the assignment of the strain to the *Arthrospira* genus, presenting similarity to the available genomes between 92.9% and 99.4% based on ANI and between 48.1% and 86.1% based on *is*DDH (Supplementary Table S1). Moreover, the presented results for all genomes were consistent with *Arthrospira* division into two main clusters based on core protein phylogeny and ITS sequence [30].

### 3.3. General Features of the Arthrospira sp. O9.13F Genome

The genome of *Arthrospira* sp. O9.13F is deposited in the GenBank database under accession number PKGD00000000, bioproject PRJNA384118. The draft genome of *Arthrospira* sp. O9.13F consisted of 928 scaffolds of a total length of 4 945 448 bp and a G + C content of 44.47%. A total of 5355 genes were annotated of which 4688 (87.5%) were protein-coding genes, 625 (11.67%) were pseudogenes and 42 (0.78%) genes were encoding RNA and three CRISPR clusters (Table 2). A total of 3278 (69.92%) of predicted genes were assigned to COGs. Most genes were assigned to protein families responsible for replication, recombination and repair as well as signal transduction. The distribution of genes into COGs is presented in Table 3. The additional annotation of the genome with the KEGG Automatic Annotation Server (https://www.genome.jp/kegg/ (accessed on 3 January 2019)) ascribed 1287 genes into 189 pathways, the highest numbers of genes were mapped to the following processes: ABC transporters (55), porphyrin and chlorophyll metabolism (41), photosynthesis (40), purine metabolism (39), two-component system (33), pyrimidine metabolism (32) and oxidative phosphorylation (31).

### 3.4. Comparative Genome Analyses

A comparative analysis of the *Arthrospira* sp. O9.13F genome with other *Arthrospira* genomes revealed the absence of genetic determinants related to the synthesis of cyanobacterial toxins, such as microcystins, nodularin or cylindrospermopsin [89]. The secondary metabolites analysis of the *Arthrospira* sp. O9.13F genome revealed the presence of five different secondary metabolite gene clusters that were present in all of the studied *Arthrospira* genomes: four cyanobactin/bacteriocin gene clusters and one phytoene synthase. The presence of phytoene is obvious because there are different carotenoids in the *Arthrospira* cells, and a complete pathway for carotenoids synthesis was present within the genome of this cyanobacterium. The secondary metabolites analysis did not indicate the presence of siderophore biosynthetic gene clusters, which agreed with the negative results of the CAS plate assay [90].

The sequences of genes involved in gliding motility present in *A. platensis* C1 and *A. platensis* NIES-39 were compared with the *Arthrospira* sp. O9.13F genome. Genes encoding the S-layer gliding motility protein (oscillin) [91], as well as genes encoding type IV pilus-related proteins involved in twitching motility (*pilA*, *pilC*-like, *pilD* and *pilT2* genes) are present in the *Arthrospira* sp. O9.13F genome.

The buoyancy of *Arthrospira* sp. O9.13F is regulated by gas vesicles (Figure 2F) encoded by certain identified genes: *gvpA*, *gvpC*, *gvpK*, *gvpFL*, *gvpV*, *gvpW*, *gvpN* and *gvpJ*. GvpA proteins form the vesicle core, while GvpC proteins provide structural support and influence the shape of the gas vesicles [92]. The proteins GvpK, GvpFL and GvpN possibly stabilise the structure of the gas vesicle. The GvpJ protein is considered to determine the shape of the gas vesicle. The functions of the GvpV and GvpW proteins remain unknown [92,93].

No plasmid sequences were observed during the DNA extraction and analysis of the genomic data for the *Arthrospira* sp. O9.13F isolate.

One incomplete prophage region was identified in the *Arthrospira* genome of 10.6 kb, with 10 total proteins, including seven phage hits and three hypothetical proteins. All 10 proteins are common in other *Arthrospira* genomes. The phage with the highest protein similarity rate to the identified region was *Citrobacter* phage Margaery (NC 028755.1).

We checked the existence of loci coding RM and CRISPR/Cas systems in the genome of the analysed cyanobacterium. The genome sequence of the *Arthrospira* sp. O9.13F strain contains three sets of the type I R-M systems (hsdM, hsdR and hsdS genes), and isoschizomers of Hin1I, Eco88I, Mph1103I, BshNI and Pfl23II complete the type II R-M systems. Genes encoding two isoschizomers of the type II solitary methyltransferases, namely, BspPI and Bsp143I, were also identified.

A total of 26 different CRISPR/Cas system direct repeats (DR) sequences were found in the *Arthrospira* sp. O9.13F genome, with six confirmed and 20 questionable. It also contains CRISPR/Cas essential *cas1* and *cas2* genes as well as the *cas6* gene and CRISPR-associated protein *csx3* gene. Repeat Associated Mysterious Protein (RAMP) genes, involved in CRISPR/Cas defence processes, were also identified, namely, *cmr2*, *cmr4*, *cmr6*, *csm1*, *csm2*, *csm3*, *csm4* and *csm5*. The organisation of the CRISPR/Cas operons is shown in Supplementary Figure S8.

#### 3.4.1. Genes Responsible for the Stress Adaptation

Cyanobacteria of the *Arthrospira* genus are known to be resistant to temperatures up to 40 °C, alkaline pH and high salt concentration. Numerous genes responsible for their adaptation capacity have been detected in the genomes of these cyanobacteria. So far, the genes encoding Na^+^/H^+^ antiporters, CO_2_ uptake (NDH^−1^) as well as genes responsible for the biosynthesis of three compatible solutes (sucrose, trehalose, glucosylglycerol or glucosylglycerate) have been described [15,94,95]. To verify whether the Siberian strain O9.13F differs from tropical ones, the presence of 68 genes related to the stress response was checked in its genome (Supplementary Table S3). We confirmed the presence of 60 genes. The sequences of these genes are almost identical or differ only in single polymorphisms between O9.13F and other *Arthrospira* strains. It is worth emphasising that the sequences of the Siberian strain that persists during the winter period in a freezing lake are the same as for the tropical strains. What is more, it should be noted that the absence of eight genes in the genome of the O.M13F strain does not necessarily mean that some pathways are incomplete because the sequence of its genome is not closed, and its size of approximately 4.95 Mbp is smaller than in the case of most *Arthrospira* genomes (from 5.75 to 6.78 Mbp) that are available in the GenBank database (Supplementary Table S2).

#### 3.4.2. *Arthrospira* sp. O9.13F Unique Genes

A comparative genomic analysis of *Arthrospira* sp. O9.13F and other *Arthrospira* genomes revealed 24 genes that are unique for this strain (Table 4). Among them, we found 14 genes encoding hypothetical proteins, RNA-binding S4 domain-containing protein as well as two genes encoding four helix bundle proteins. Moreover, unique CDs encode proteins involved in the immunity system, N-6 DNA methylase, which is most similar with Methylase AflIII, the DUF433 domain-containing protein that might be a part of the putative toxin/antitoxin system ([96]) as well as the WYL domain-containing protein, which is a regulator of the Type VI-D CRISPR-Cas system [97] and thus might regulate the response to environmental stresses [98] (Table 4).

## 4. Discussion

The temperature of the water in Solenoye Lake varies between 0 and 30 °C during vegetation season. The water salinity level varies from mesohaline during spring to polyhaline in the remaining seasons, and seasonal pH variation is in the range of 8.94–9.94 [7]. The lake is located on salt clay, and it freezes only on the surface during the winter. The ice cover remains for three months, from December to the beginning of March. The surface water temperature in these months ranges between −3 °C and −15 °C, with an average of approximately −10 °C. In winter, brine, which has a large heat accumulation capacity, may keep a higher temperature on the lower water layers. In addition, the salinity of the water lowers the temperature of the ice cover formation. The ice formation on the lake’s surface increases salinity in the lower water layers and allows the medium to remain liquid [99]. The cyanobacteria blooms in Solenoye Lake are observed annually. The composition of the lake’s phytoplankton has been investigated since 2007 [7]. During the entire ice-free period, cyanobacteria abundantly develop in the lake as does the species of the *Arthrospira* genus. *Arthrospira* forms the bloom in the warmer season, although its vegetation is noted in the lake even in the winter under the ice. The presence of *Arthrospira* in Solenoye Lake was noted even a few decades ago when there was a resort where the healing properties of the microflora in the lake were used to treat a variety of human diseases [7].

This study is the first comprehensive, physiological and genomic description of the *Arthrospira* strain O9.13F isolated from Solenoye Lake in September 2013. It is the first well-characterised isolate of *Arthrospira* found in Siberia. The described strain O9.13F is cultivable in laboratory conditions and showed the highest relative growth index and highest accumulation of proteins and photosynthetic pigments in the biomass upon cultivation at 20 °C. The observed optimal growth temperature is significantly lower than the temperature of 35 °C reported in the literature as optimal for most *Arthrospira* strains that typically originate from tropical areas. The earlier study of Kumar et al. 2011 showed that tropical strains of *Arthrospira* could not effectively grow and accumulate biopigments at 20 °C. We obtained similar observations during our research. Two reference strains, PCC 8005 and PCC 7345, grew less efficiently at 20 °C than the Siberian strain (Figure 4). While at the temperature of 15 °C, both strains stopped their metabolism, and filaments started to fall apart; therefore, the measurements of proteins and fatty acids were below the sensitivity limit of the method.

Nevertheless, the O9.13F strain was able to grow at 15 °C. Interestingly, we observed the highest growth index in the Zarrouk medium at this temperature with the most elevated tested salinity of 200 g/L. Furthermore, we showed that *Arthrospira* O9.13F could persist in temperatures around 9 °C for several months. They were unable, however to survive freezing at −20 °C in laboratory conditions. The waters of Lake Solenoje do not freeze to the bottom. Considering the properties of the O9.13F strain and the fact that in the ice-covered lake, the water in the deeper layers is more saline and warmer than those closer to the surface [99], we can hypothesise that the Siberian *Arthrospira* strain can survive the winter periods on the bottom of the lake and cause annual blooms observed in the summer.

The fatty acid content of the biomass of the Siberian strain does not deviate much from the biomass of two other analysed strains originating from tropical climates when each was cultivated in their optimal temperatures: 20 °C for O9.13F and 28 °C for PCC 8005 and PCC 7345. The same trend of a decrease of gamma-linolenic acid in favour of palmitic acid caused by a change of the temperature was observed for the Siberian strain as for the tropical ones. A drop in temperature from 30 to 20 °C led to a decrease in palmitic acid accompanied by an increase in linoleic, and the gamma-linolenic acid content was observed earlier by [100], suggesting an increase in membrane fluidity in response to the temperature change. Colla et al. [101] observed that elevation of the growth temperature from 30 °C to 35 °C increased the palmitoleic and linoleic acid concentration but decreased gamma-linolenic acid. Thus, the observed reduced amount of gamma-linolenic acid in the biomass of the Siberian strain after the elevation of the growth temperature from 20 °C to 28 °C confirm its acclimation to cooler than tropical climate.

Additionally, we concluded that it is possible to recover the *Arthrospira* strains after long incubation periods at a low temperature (9 °C) and in low light conditions (10 µmol photons m^−2^ s^−1^). This observation is valuable in case of storage problems with this species in culture collections as the *Arthrospira* strains are known to not survive the cryopreservation and lyophilisation procedures [102].

Based on this study’s detailed genetic characteristics, the newly isolated Siberian strain O9.13F belongs to the *Arthrospira/Limnospira* genus. On the genomic level, the Siberian strain O9.13F is highly similar to 17 other *Arthrospira/Limnospira* genomes that are available in the GenBank. Undoubtedly it is not a new species, and based on genomic analyses it belongs to the group gathering strains *A. maxima* CS-328, *A. platensis* C1, *Arthrospira* sp. PCC 8005, TJSD091, TJSD092, BM1 and *Arthrospira fusiformis* KN and SAG 85.79. The second group consists of the strains *A. platensis* Paraca, NIES-39, NIES-46, YZ, FACHBB-835, FACHBB-439, FACHBB-971 and *Arthrospira* sp. PCC9108, PLM2.Bin9 and SH-MAG29. Presently published *Arthrospira* genomes belong to the I.A, I.B and II.A ITS subclusters distinguished by Baurain et al. The strain *Arthrospira* sp. O9.13F presented in this work is not only the first strain isolated from a climate other than a tropical or subtropical one (Siberia, Russia) but is also a representative of a new clade III based on the 16S rRNA gene (according to Comte et al. 2013), for which the genomic sequence is available in public databases (PKGD00000000).

What is more, here we report that the Siberian strain O9.13F has two different ITS variants in contrast to most *Arthrospira* strains that possess only one variant of the ITS within the ribosomal operon, except for strain PCC 7345 that was shown by [30].

The discrepancy between the phylogenies based on ITS and *cpcBA* has already been observed in the works of [5,32], in which the possibility of recombination has been postulated. Our results of the *cpcBA* sequence analysis do not agree with the data presented by [31,34]. These authors only used the noncoding IGS fragment between the *cpcBA* genes. It could be caused by the fact that we used, apart from the IGS region, also fragments of the flanking *cpcB* and *cpcA* genes, similar to Manen and Falquet 2002 and Dadheech et al., 2010 earlier [5,32].

The results of the phylogenetic analyses based on the 16S rRNA gene, ITS and phycocyanin operon fragment *cpcBA* are not consistent, indicating that these three genetic markers, which are typically used in the phylogenetic analysis of cyanobacteria, are not appropriate for the classification of the new isolates of *Arthrospira*. They do not allow for establishing a taxonomy of this genus as their results are not congruent. Furthermore, the position of the PCC9901 strain, which represents the 16S clade III, is undetermined.

Nevertheless, the results obtained in this study reveal the presence of only two groups within the genus *Arthrospira*. Finally, the strain O9.13F constantly merges with the same group of strains from the first clad 16S and the first ITS cluster. And these observations correlate with the results of genomic analyses, ANI, *is*DDH and core protein-based phylogeny.

The genomic comparisons showed that the O9.13F strain, like other members of this genus, possesses extensive R-M and CRISPR/Cas systems that enhance their protection system against exogenic invasive genetic elements, such as plasmids and viruses as well as intragenomic rearrangements. In addition, the gas vesicle genes operon of strain O9.13F is similar to other *Arthrospira* strains. The virtual analysis agreed with the TEM analysis, and both confirmed that the Siberian strain produces gas vesicles to regulate buoyancy depending on the light intensity.

Comparative genomics revealed no apparent characteristic genes that may be directly responsible for the adaptation of the Siberian strain to low temperatures of its habitat during the winter period. However, among unique CDs, we found those which encode proteins that might regulate the response to environmental stresses, such as methylase, the putative toxin/antitoxin system or a regulator of the CRISPR-Cas system [96,97,98]. What is more, strain O9.13F did not possess a different set of genes associated with the stress response than tropical strains.

The observed discrepancy between different physiological characteristics (observed acclimatisation) with the simultaneous lack of significant genetic differences (no signs of adaptation in the form of unique genes or different genes conditioning the survival of the low-temperature period) remains an intriguing observation that requires further research. Furthermore, in further studies aimed at explaining the mechanisms that allow the Siberian strain to survive the winter period, the influence of other microorganisms that coexist in the waters of Lake Solonye should be considered.

Considering all the data we collected; we can conclude that the genus *Arthrospira* has a much broader acclimation capacity than previously reported. The observed physiological properties of the Siberian strain indicate their usefulness in terms of production in temperate climate zones with the use of local water resources, such as water from the Baltic Sea.

## Figures and Tables

**Table 1:** Total FA composition (%) of *Arthrospira* strains O9.13F, PCC 7345 and PCC 8005 cultivated at 28 °C.

Fatty Acid	O9.13F	PCC 8005	PCC 7345
16:0	36.71 ± 3.93	30.81 ± 1.2	31.64 ± 3.87
16:1	8.80 ± 1.1	10.29 ± 0.65	9.97 ± 1.13
18:0	1.12 ± 0.33	0.87 ± 0.16	1.62 ± 0.68
18:1n9c	2.40 ± 0.97	2.59 ± 0.32	1.54 ± 0.66
18:1n11c	1.67 ± 0.3	1.29 ± 0.04	1.32 ± 0.35
18:2n6c	18.86 ± 0.34	18.78 ± 0.9	19.66 ± 2.43
18:3n6	30.45 ± 4.45	35.37 ± 1.98	34.26 ± 6.16

*n* = 3; 16:0—Palmitic acid; 16:1—Palmitoleic acid; 18:0—Stearic acid; 18:1n9c—Oleic acid; 18:1n11c—Vaccenic acid; 18:2n6c—Linoleic acid; 18:3n6—γ-Linolenic acid. The data are means ± standard deviations based on three replicate analyses.

**Table 2:** Genome statistics of *Arthrospira* sp. O9.13F.

Attribute	Value	% of Total
Genome size (bp)	4,945,448	100
DNA coding (bp)	3,759,108	76.01
DNA G + C (bp)	2,198,162	44.5
DNA scaffolds	928	-
Total genes	5355	100
Protein coding genes	4688	87.5
RNA genes	42	0.78
Pseudogenes	625	11.67
Genes in internal clusters	-	-
Genes with function prediction	3363	62.80
Genes assigned to COGs	3278	69.92
Genes with Pfam domains	3237	69.05
Genes with signal peptides	400	8.53
Genes with transmembrane helices	862	18.39
CRISPR repeats	3	0.06

**Table 3:** The number of genes associated with general COG functional categories.

Code	Value	% of Total *	Description
J	146	4.45	Translation, ribosomal structure and biogenesis
A	0	0	RNA processing and modification
K	145	4.42	Transcription
L	311	9.49	Replication, recombination and repair
B	2	0.06	Chromatin structure and dynamics
D	25	0.76	Cell cycle control, cell division, chromosome partitioning
V	84	2.56	Defence mechanisms
T	308	9.4	Signal transduction mechanisms
M	209	6.38	Cell wall/membrane biogenesis
N	39	1.19	Cell motility
U	44	1.34	Intracellular trafficking and secretion
O	156	4.76	Posttranslational modification, protein turnover, chaperones
C	174	5.31	Energy production and conversion
G	140	4.27	Carbohydrate transport and metabolism
E	195	5.95	Amino acid transport and metabolism
F	58	1.77	Nucleotide transport and metabolism
H	115	3.51	Coenzyme transport and metabolism
I	73	2.23	Lipid transport and metabolism
P	138	4.21	Inorganic ion transport and metabolism
Q	95	2.90	Secondary metabolites biosynthesis, transport and catabolism
R	499	15.22	General function prediction only
S	322	9.82	Function unknown
-	1811	33.82	Not in COGs

* The % of total is based on the total number of protein coding genes in the genome.

**Table 4:** *Arthrospira* sp. O9.13F-specific CDs.

	ORF	Description	The Most Similar Sequence in Genbank	Identity
1	B9S53_26655	hypothetical protein	-	-
2	B9S53_26345	hypothetical protein	AI-2E family transporter [*Natronoflexus* pectinivorans] WP_165921875.1	61%
3	B9S53_26040	IS91 family transposase	transposase [*Lentisphaerae* bacterium] MBM4144559.1	73%
4	B9S53_26035	hypothetical protein	WYL domain-containing protein [*Lentisphaerae* bacterium] MBM4165248.1	83%
5	B9S53_25925	hypothetical protein	-	-
6	B9S53_25920	RNA-binding protein	RNA-binding S4 domain-containing protein [*Flavobacteriales* bacterium] TVQ76998.1	78%
7	B9S53_25790	hypothetical protein	-	-
8	B9S53_25595	four helix bundle protein	four helix bundle protein [*Chloroflexi* bacterium] HFC09004.1	67%
9	B9S53_25590	hypothetical protein	four helix bundle protein [*Chloroflexi* bacterium] HFC09004.1	53%
10	B9S53_25580	four helix bundle protein	-	
11	B9S53_24895	hypothetical protein	N-6 DNA methylase [*Sphaerospermopsis* sp. FACHB-1094] WP_190648063.1	59%
12	B9S53_23130	hypothetical protein	ROK family protein [*Pseudanabaena* sp. FACHB-1277] WP_190353199.1	43%
13	B9S53_22765	hypothetical protein	-	-
14	B9S53_21915	hypothetical protein	-	-
15	B9S53_21010	IS4 family transposase	transposase [*Phormidium* sp. FACHB-1136] WP_199324687.1	75%
16	B9S53_20985	hypothetical protein	-	-
17	B9S53_20435	hypothetical protein	hypothetical protein [*Cylindrospermopsis* raciborskii] WP_071249329.1	67%
18	B9S53_19635	hypothetical protein	-	-
19	B9S53_12695	hypothetical protein	-	-
20	B9S53_12545	hypothetical protein	-	-
21	B9S53_11425	hypothetical protein	aldolase [*Chloroflexi* bacterium] MBT7081734.1	50%
22	B9S53_11330	IS4 family transposase	transposase, partial [*Leptolyngbya* sp. PCC 6406] WP_155834780.1	75%
23	B9S53_06535	DUF2281 domain-containing protein	DUF2281 domain-containing protein [*Geitlerinema* sp. P-1104] NMG59147.1	94%
24	B9S53_01945	hypothetical protein	hypothetical protein [*Nodularia* sp. LEGE 06071] WP_193996843.1	32%

## Data Availability

The data that support the findings of this study are available from the corresponding authors (K.F.W. and M.W.), upon request. The draft genome sequence of *Arthrospira* sp. O9.13F has been deposited in NCBI/Genbank under accession number PKGD00000000. The nucleotide sequence data reported in this paper are available under the following accession numbers: 16S rDNA; MF509165, ITS; MF509166–MF509169, *cpcBA* and MF509119.

## Supplementary Materials

The following are available online at www.mdpi.com/article/10.3390/cells10123411/s1, Figure S1: The cyanobacterial bloom, Lake Solenoye, Figure S2: The growth index quantity of chlorophyll, total carotenoids extracted from biomass of *Arthrospira* sp. O9.13F cultivated at different temperatures and salt concentrations, Figure S3: Acclimation of *Arthrospira* strains to long-term cold stress, Figure S4: The percentage of the total fatty acids in the biomass of *Arthrospira* sp. O9.13F cultivated at 20, 28 and 37 °C, Figure S5: Phylogenetic tree of *Arthrospira* strains based on 16S rRNA partial sequences, Figure S6: Phylogenetic tree of *Arthrospira* strains based on ITS rRNA partial sequences, Figure S7: Phylogenetic tree of *Arthrospira* strains based on *cpcBA* sequences, Figure S8: Organisation of CRISPR/Cas operons in *Arthrospira* sp. O9.13F genome, Table S1. ANI and *is*DDH values between *Arthospira sp.* O9.13F and other 16 *Arthrospira* strains for which genomes are available in the GenBank, Table S2: Summary of statistics of *Arthrospira* genomes available on NCBI. Table S3: Presence of genes responsible for the stress adaptation in *Arthrospira* genomes. References [103,104] are refered to in Supplementary Materials.
