# Supplementary material for: Comparative Genomics and Physiological Investigation of a New Arthrospira/Limnospira Strain O9.13F Isolated from an Alkaline, Winter Freezing, Siberian Lake
Source: Cells. 2021 Dec 3;10(12):3411. doi: 10.3390/cells10123411 (PMC8700078; doi:10.3390/cells10123411)
Supplement: Supplementary file 1 [file cells-10-03411-s001.zip › cells-1444419-supplementary.pdf]

Supplementary Materials

Comparative Genomics and Physiological Investigation of a New *Arthrospira/Limnospira* Strain O9.13F Isolated from an Alkaline, Winter Freezing, Siberian Lake

Agnieszka E. Misztak [1,‡], Malgorzata Waleron [1,*,†], Magda Furmaniak [3], Michal M. Waleron [3], Olga Bazhenova [4], Maurycy Daroch [5] and Krzysztof F. Waleron [3,*]

[1] Laboratory of Plant Protection and Biotechnology, Intercollegiate Faculty of Biotechnology UG and MUG, University of Gdansk, 80-307 Gdansk, Poland; amisztak@uliege.be

[2] Department of Pharmaceutical Microbiology, Faculty of Pharmacy, Medical University of Gdansk, 80-416 Gdansk, Poland; magda.furmaniak@acteryon.com (M.F.); michal.waleron@gumed.edu.pl (M.M.W.)

[3] Department of Ecology, Nature Management and Biology, Omsk State Agrarian University Named after P.A. Stolypin, 644008 Omsk, Russia; olga52@bk.ru

[4] School of Environment and Energy, Peking University Shenzhen Graduate School, Shenzhen 518055, China; m.daroch@pkusz.edu.cn

* Correspondence: malgorzata.waleron@biotech.ug.edu.pl (M.W.); krzysztof.waleron@gumed.edu.pl (K.F.W.)

† Equal contribution.

‡ Current address: Unit of Animal Genomics, GIGA Institute, University of Liège, 4031 Liège, Belgium

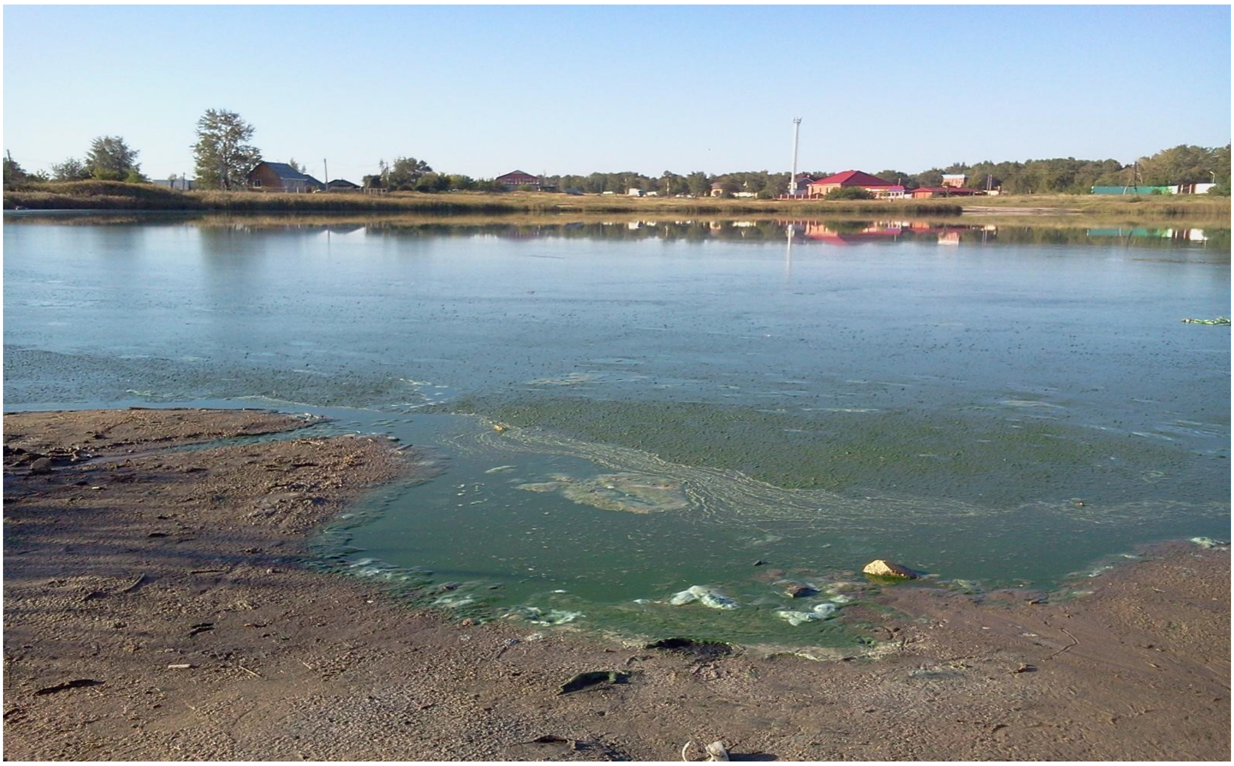

Figure S1. Lake Solenoye in Omsk, Russia (54°53'11.3"N 73°20'51.7"E). The cyanobacterial bloom visibly washes off-shore, 11 September 2013.

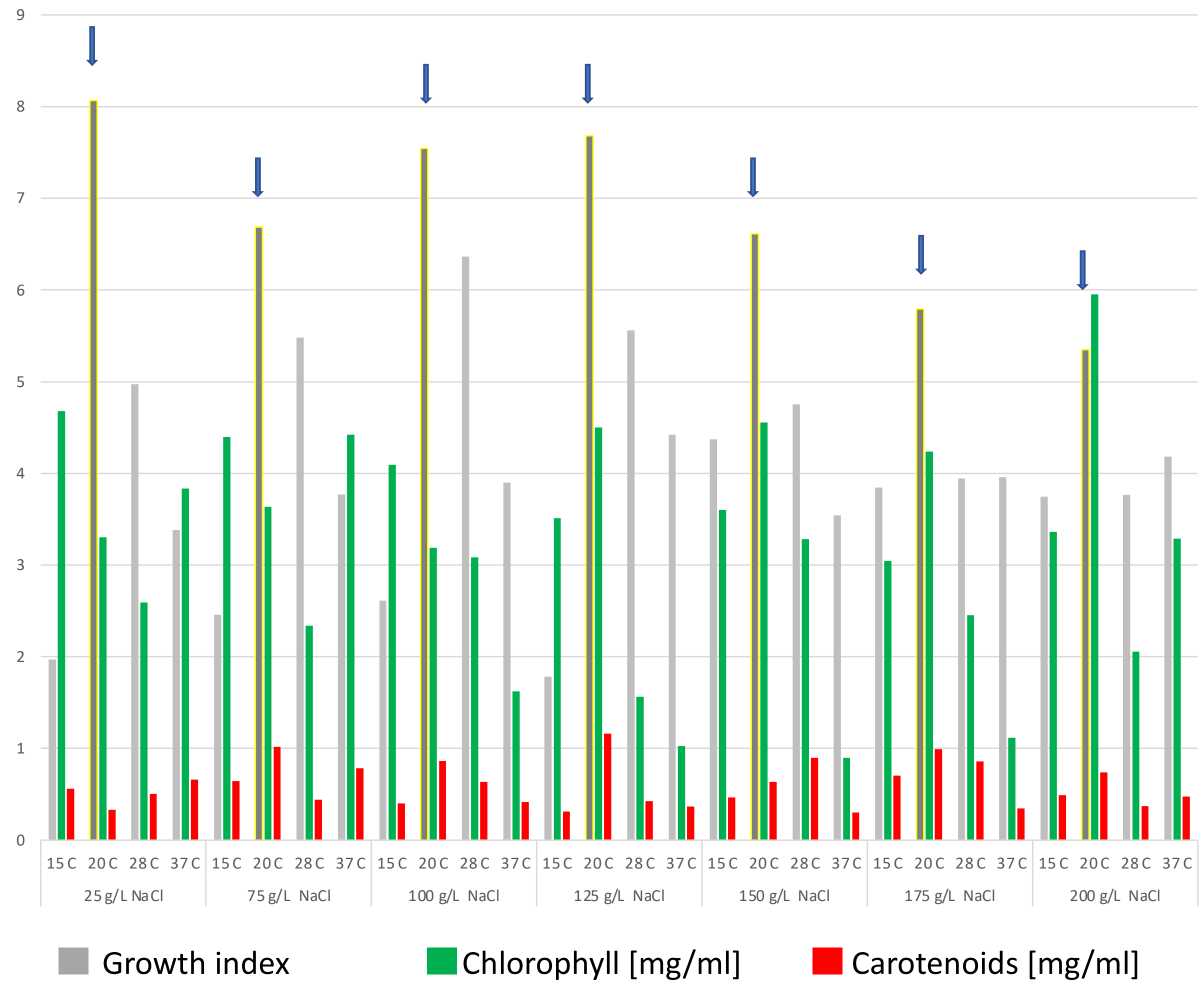

Figure S2. The growth index and quantity of chlorophyll and total carotenoids [mg/ml] extracted from biomass of *Arthrospira* sp. O9.13F cultivated at 15, 20, 28 and 37 °C in media containing different NaCl concentration: 25 g/L, 75 g/L, 100 g/L, 100 g/L, 125 g/L, 150 g/L, 175 g/L, 200 g/L. The growth rate factor at 20 have been marked with blue arrows.

Strain	Control Magnification 400x	Microscopy after the 10-month experiment Magnification 400x	Recovery of cultures on solid medium after 10-month experiment *
SAG 49.88	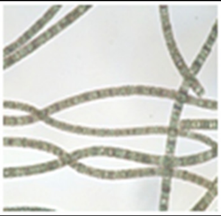	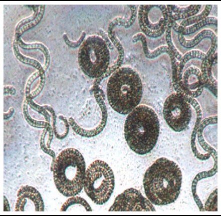	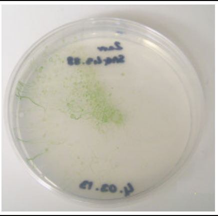
PCC 8005	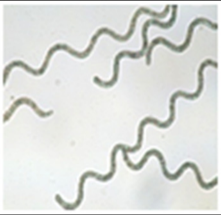	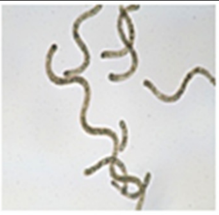	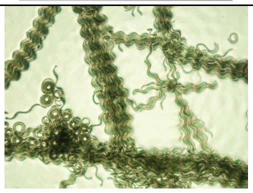 100x magnification.
CCALA 23	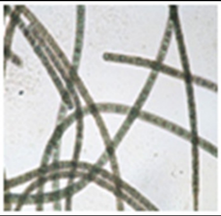	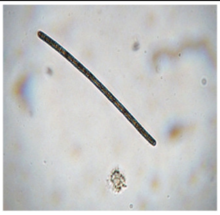	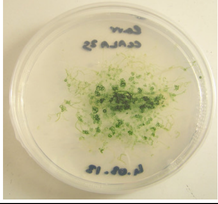
PCC 7345	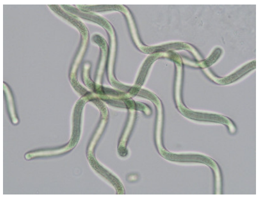	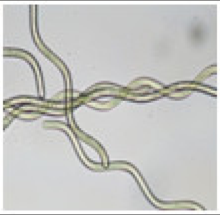	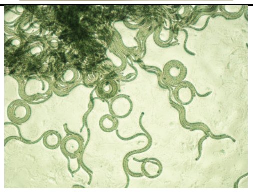 100x magnification.
O9.13F	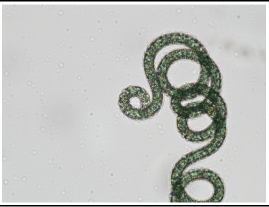	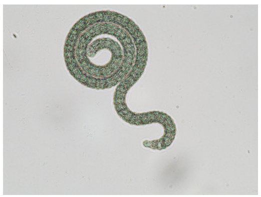	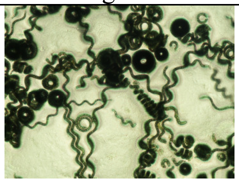 100x magnification.

Figure S3. Acclimation of *Arthrospira* strains to long-term cold stress

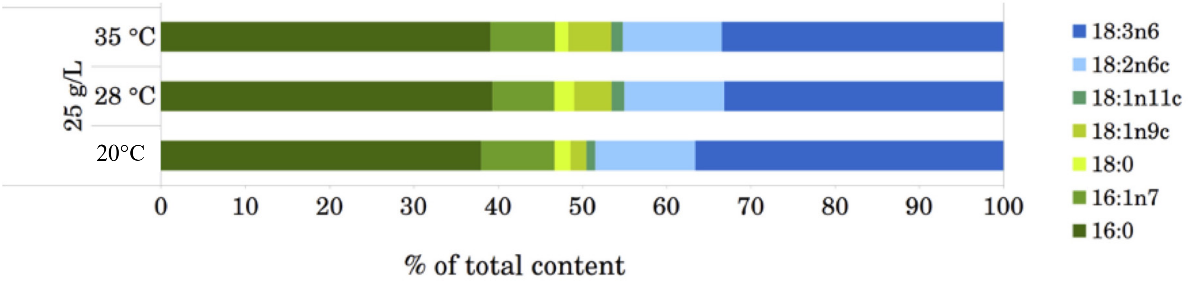

Figure S4. The percentage of the total fatty acids in the biomass of *Arthrospira* sp. O9.13F cultivated at 20, 28 and 37 °C. There were significant differences ($p<0.05$) between individual fatty acids levels extracted from biomass grown under conditions in all except stearic and linolenic acids. Palmitic acid (16:0), gamma-linolenic acid (18:3n6), linoleic acid (18:2n6c), oleic acid (C18:1n9c), cis-vaccenic acid, stearic acid (C18:0), palmitic acid (C16:0) and palmitoleic acid (16:1).

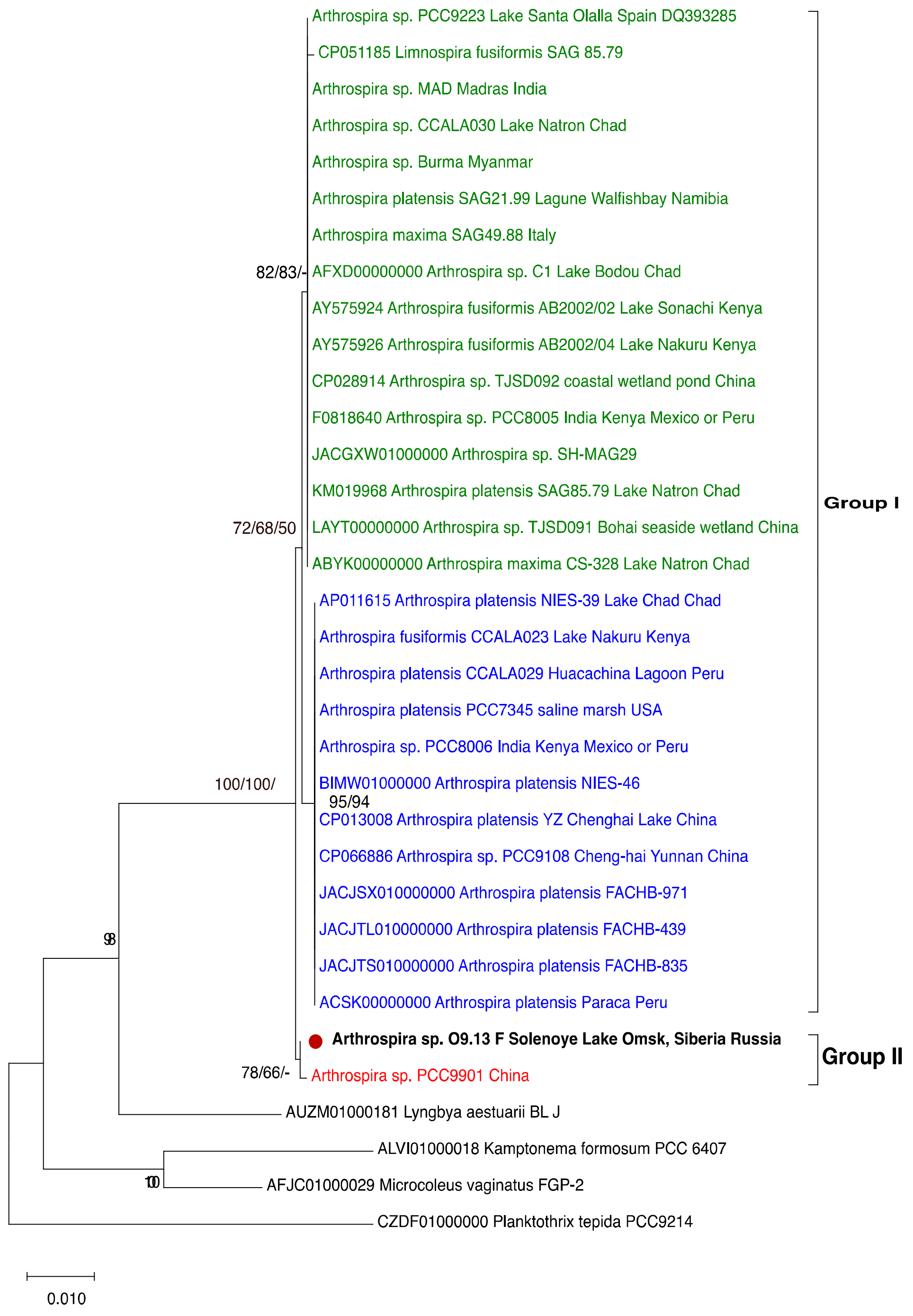

Figure S5 Phylogenetic tree of *Arthrospira* strains based on 16S rRNA partial sequences (1102 bp) reconstructed using the Maximum-likelihood (ML) analysis. Numbers above branches indicate the bootstrap value (as percentages of 1000 replications) for ML, NJ and MP methods present study are indicated in bold font Sequence of strain O9.13F is indicated in bold font. Colors are corresponding to 16S clads: breen -clade 1, blue – clade II (according to [30]) and red - clade III (according to [88]).

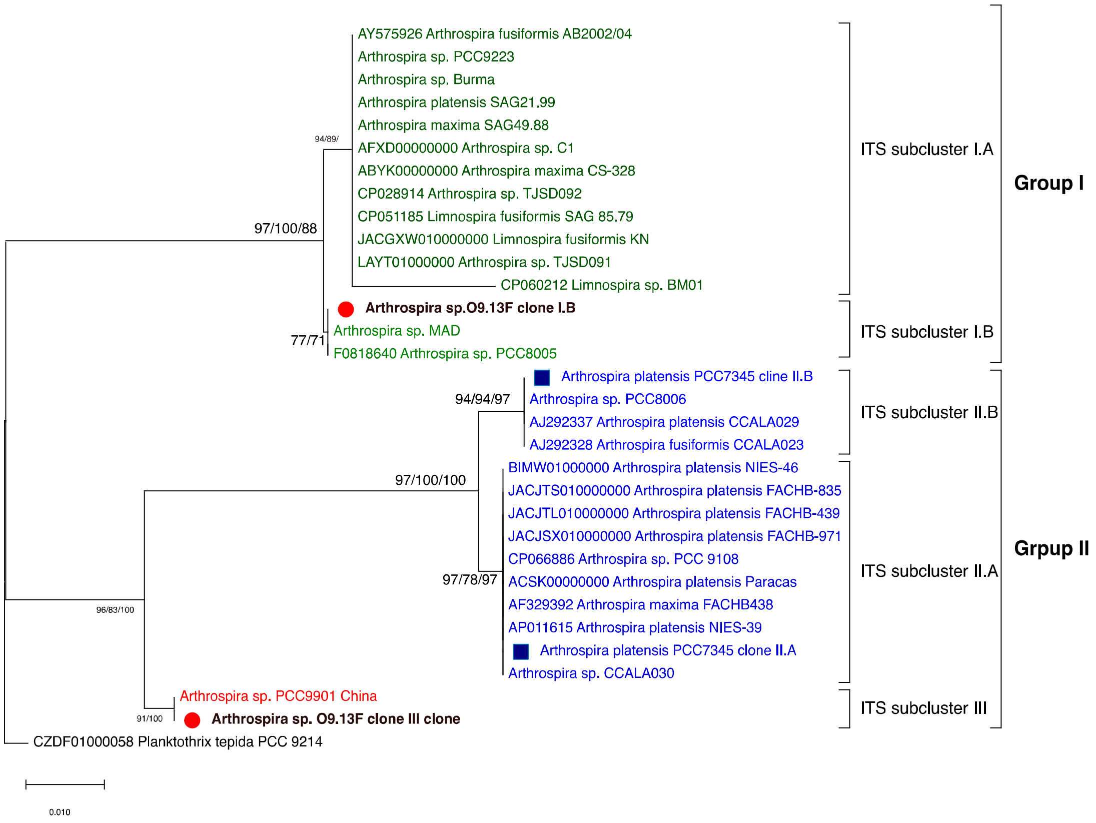

Figure S6 Phylogenetic tree of *Arthrospira* strains based on ITS rRNA partial sequences (470 bp) reconstructed using the Maximum-likelihood (ML) analysis. Numbers above branches indicate the bootstrap value (as percentages of 1000 replications) for ML, NJ and MP methods Sequence of strain O9.13F is indicated in bold font. Colors are corresponding to 16S clads: breen -clade 1, blue – clade II (according to [30]) and red - clade III (according to [88]). Two genetic variants of ITS sequences from strains O9.13F and PCC7345 were marked with red dots and blue squares, respectively.

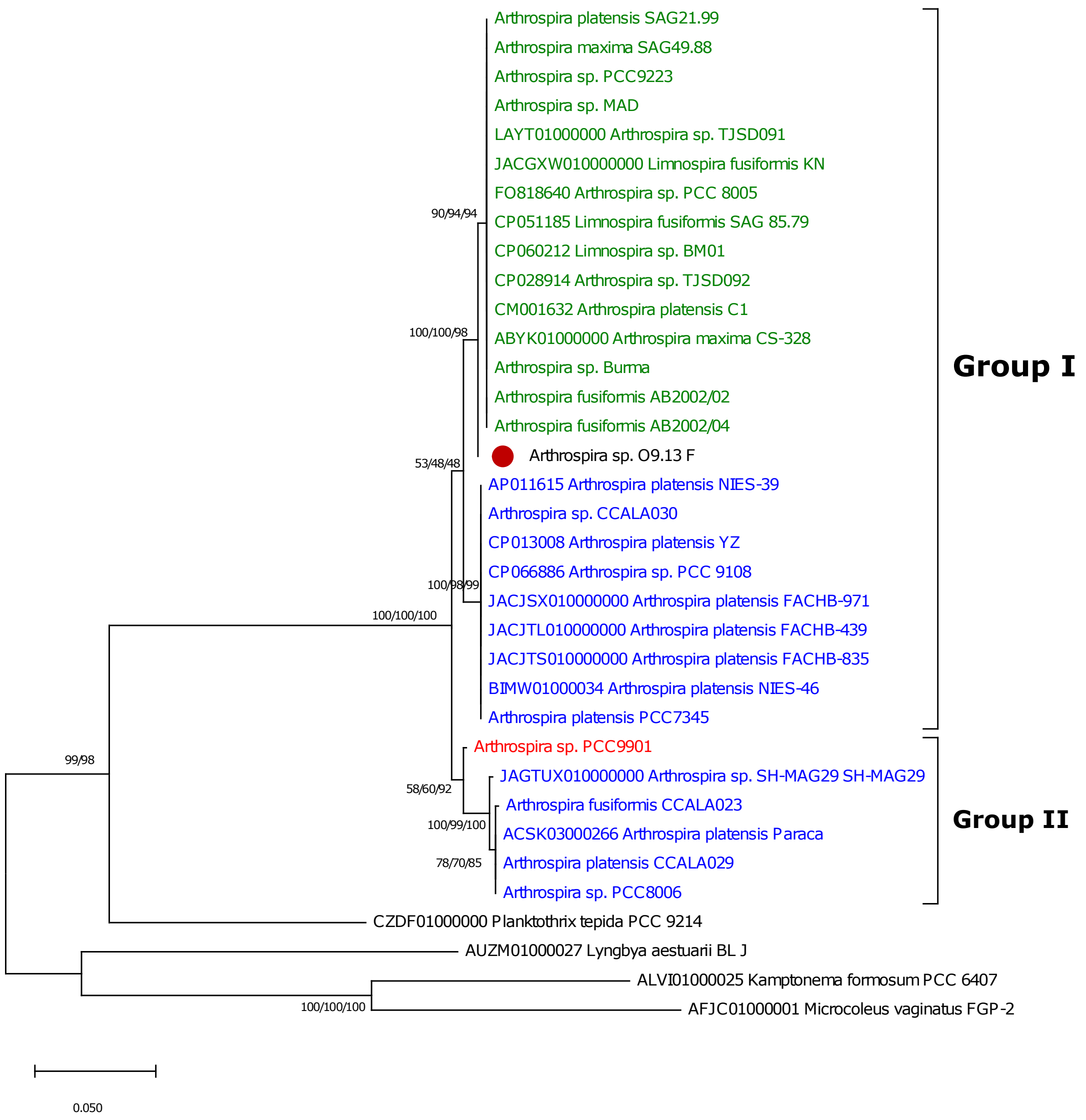

Figure S7. Phylogenetic tree of *Arthrospira* strains based on *cpcBA* sequences (865 bp) reconstructed using the Maximum-likelihood (ML) analysis. Numbers above branches indicate the bootstrap value (as percentages of 1000 replications) for ML, NJ and MP methods Sequence of strain O9.13F is indicated in bold font. Colors are corresponding to 16S clads: breen -clade 1, blue – clade II (according to [30]) and red - clade III (according to [88]).

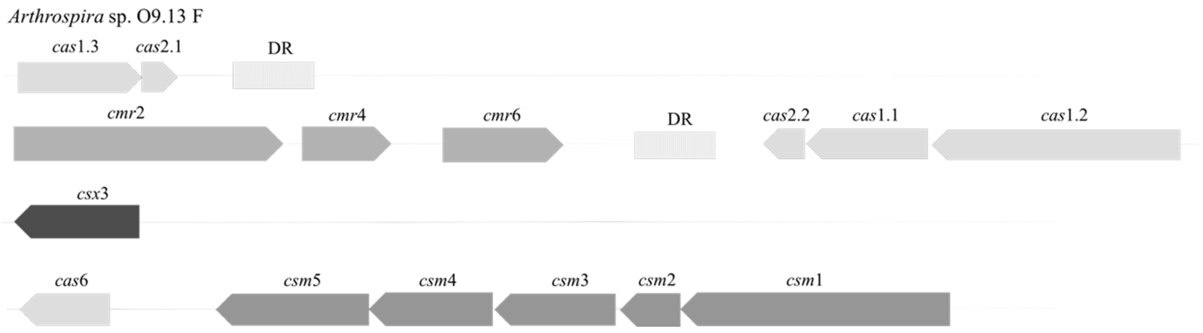

Figure S8 Organization of CRISPR/Cas operons in *Arthrospira* sp. O9.13F genome; DR - stands for Direct Repeats.

Table S1. ANI and *is*DDH values between *Arthospira sp.* O9.13F and other 16 *Arthrospira* strains for which genomes are available in the GenBank. The lower triangle shows the isDDH values, and the upper triangle displays ANI values. The sequence of *Arthospira sp.* O9.13F is marked the boldface;

	Genus	Strain	Genbank Accession No	*is* DDH 1	2	3	4	5	6	18	19	7	8	9	10	11	12	13	14	15	16	17	ITS cluster
1	***Arthrospira* sp.**	**O9.13F**	**PKGD00000000**	**-**	**81.5**	**86.1**	**85.6**	**86**	**85.8**	**86.6**	**87**	**85.5**	**74**	**75.2**	**53.7**	**50.1**	**51.5**	**50.5**	**49.7**	**51.7**	**48.5**	**47.8**	**I**
2	*Arthrospira* sp.	PCC8005	FO818640	**98.9**	-	90.2	92.2	90.4	89.8	95.2	91.1	89.9	85.6	87.3	53.8	50.3	51.9	50.8	50	51.9	48.2	47.2	I
3	*Arthrospira* sp.	TJSD091	LAYT00000000	**98.7**	99.2	-	75.7	92.4	92.2	93	93	91.7	83.3	84.5	53.9	50.6	52.3	51.3	50.4	52	48.5	47.5	I
4	*Arthrospira* sp.	TJSD092	CP028914	**98.7**	99.2	99.8	-	92.6	92	93.1	92.7	91.7	83.9	85.8	53.8	50.2	51.9	50.9	50	51.9	48.1	47.1	I
5	*Arthrospira maxima*	CS-328	ABYK00000000	**98.7**	99.2	99.4	99.4	-	92.6	93.5	92.9	92.9	82.7	84.1	54	50.4	52	50.9	49.9	51.9	48.1	47.3	I
6	*Arthrospira platensis*	C1	AFXD00000000	**98.6**	99.1	99.4	99.4	99.4	-	92.7	92.4	91.8	82.7	83.7	53.8	50.2	51.8	50.6	49.7	51.7	48.2	47.4	I
7	*Arthrospira* sp.	BM01	CP060212	**98.73**	99.0	97.61	99.27	99.37	99.31	-	93.8	92.7	54.4	56	54.1	50.7	52.3	51.1	50.3	52.1	48.2	47.2	I
8	*Arthrospira fusiformis*	KN	JACGXW010000000	**98.66**	99.22	99.42	99.40	99.36	99.39	99.39	-	92.4	54.2	55.4	53.8	50.9	52.3	51.4	50.5	51.8	48.8	47.6	I
9	*Arthrospira fusiformis*	SAG85.79	CP051185	**98.63**	99.31	99.32	99.29	99.28	99.31	99.28	99.41	-	54.1	55.9	53.8	50.2	51.9	50.8	49.8	51.7	48.2	47.2	I
10	*Arthrospira platensis*	FACHB835	JACJTS01	**94.11**	94.14	94.19	94.20	94.18	94.12	94.16	94.19	94.18	-	99.7	97.9	93.5	98.6	97.6	95.7	98.3	82.7	64.8	II
11	*Arthrospira platensis*	FACHB439	JACJTL01	**94.38**	94.36	94.46	94.50	94.49	94.43	94.45	94.47	94.47	99.26	-	92.7	91.2	99.4	98.8	97.1	98.6	82	63.3	II
12	*Arthrospira platensis*	FACHB971	JACJSX01	**94.09**	94.15	93.49	93.30	94.18	94.14	94.15	94.15	94.16	99.80	99.21	-	92.5	91.1	91.2	89.3	92.6	77.1	64.8	II
13	*Arthrospira* sp.	PCC9108	CP066886	**93.77**	93.77	93.82	93.77	93.75	93.75	93.75	93.83	93.75	99.66	99.36	99.60	-	95.7	96.1	94.1	92.5	74.9	62.6	II
14	*Arthrospira platensis*	YZ	CP13008	**94.1**	94.08	94.2	94.2	94.09	94.07	94.08	94.15	94.10	99.43	99.23	99.40	99.54	-	93.4	91.6	66.9	73.4	61.8	II
15	*Arthrospira platensis*	Paraca	ACSK00000000	**93.9**	93.82	93.9	94	93.81	93.78	93.80	93.87	93.81	99.44	99.18	99.41	99.60	99.34	-	97.4	64.7	75.6	63.7	II
16	*Arthrospira platensis*	NIES-39	AP011615	**93.7**	93.8	93.8	93.8	93.8	93.7	93.65	93.71	93.64	99.31	99.05	99.26	99.49	99.22	99.65	-	52	76	63.4	II
17	*Arthrospira* sp.	NIES-46	BIMW00000000	**93.7**	93.8	93.8	93.8	93.8	93.7	93.74	93.77	93.74	99.30	99.03	98.97	99.53	99.3	99.5	99.8	-	79.2	66.1	II
18	*Arthrospira* sp.	PLM2Bin9	REEX00000000	**92.9**	92.9	93	93	92.9	92.9	92.91	92.94	92.94	97.78	97.51	97.53	97.95	97.97	98	98.1	98.1	-	74.0	II
19	*Arthrospira sp.*	SH-MAG29	JAGTUX01	**92.76**	92.74	92.79	92.76	92.74	92.72	92.75	92.75	92.75	96.06	95.90	96.05	96.20	96.03	96.28	96.33	96.33	97.12	-	II
				ANI																			

is DDH <70 = different species; isDDH >70<79 = species level (the same species. different subspecies); *is*DDH >79 = subspecies level [103]
ANI >96 = the same species [104].

Table S2. Summary of statistics of *Arthrospira* genomes available on NCBI

Strain	ITS cluster	Genome size [bp]	Contigs	GC [%]
PKGD00000000 *Arthrospira* sp. O9.13F	I.B / III	4,945,448	928	44.4
JACJTS00000000 *Arthrospira platensis* FACHB835	I.A	5,825,036	372	44.5
LAYT00000000 *Arthrospira* sp. TJSD091	I.A	5,978,827	359	44.8
CP051185 *Arthrospira fusiformis* SAG 85.79	I.A	6,423,694	1	44.9
CP028914 *Arthrospira* sp. TJSD092	I.A	6,434,389	1	44.9
CP060212 *Arthrospira* sp. BM01	I.A	6,228,153	1	43.9
JACGXW010000000 *Arthrospira fusiformis* KN	I.A	5,784,041	401	44.4
ABYK01000000 *Arthrospira maxima* CS-328	I.A	6,003,314	129	44.8
CM001632 *Arthrospira platensis* C1	I.A	6,089,210	1	43.5
FO818640 *Arthrospira* sp. PCC8005	I.B	6,228,153	1	44.7
JACJSX00000000 *Arthrospira platensis* FACHB971	II.A	5,624,234	315	44.6
JACJTL00000000 *Arthrospira platensis* FACHB439	II.A	5,800,128	308	44.5
JAGTUX00000000 *Arthrospira platensis* SH MAG29 *	II.A	4,854,096	346	44.4
BIMW01000001 *Arthrospira platensis* NIES46	II.A	5,728,646	343	44.5
REEX01000000 *Arthrospira* sp. PLM2Bin9 *	II.A	4,788,243	181	44.4
AP011615 *Arthrospira platensis* NIES39	II.A	6,788,435	1	43.7
ACSK03000000 *Arthrospira platensis* Paraca	II.A	6,501,886	268	44.3
CP066886 *Arthrospira* sp. PCC9108	II.A	6,763,964	1	44.2
CP013008 *Arthrospira platensis* YZ	II.A	6,520,772	1	44.2

* A Metagenome-Assembled Genome (*MAG*)

Table S3 Presence of genes responsible for the stress adaptation in *Arthrospira* genomes.

Gene *	protein	*Arthrospira* genomes with accession no.																		
		PKGD00000000 - O9.13F	FO818640 - PCC8005	CM001632 - C1	ABYK01000000 - CS-328	AP011615 - NIES39	ACSK03000000 - Paraca	LAYT01000000 - TJSD091	CP028914 - TJSD092	CP013008 - YZ	REEX01000000 - PLM2Bin9	BIMW01000000 - NIES46	CP051185 - SAG 85.79	JACGXW00000000 - KN	CP060212 - BM01	CP066886 - PCC9108	JACJTL00000000 - FACHB439	JACJTS00000000 - FACHB835	JACJSX00000000 - FACHB971	JAGTUX00000000 - FACHB835
agp	glucose-1-phosphatase/inositol phosphatase/ glucose-1-phosphate adenylyltransferase	+	+	+	+	+	+	+	+	+	+	+	+	+	+	+	+	+	+	+
aqpZ	aquaporin Z	+	+	+	+	-	-	+	+	-	+	-	-	+	+	-	-	-	-	+
asd	Aspartate-semialdehyde dehydrogenase	+	+	+	+	+	+	+	+	+	+	+	+	+	+	+	+	+	+	+
betB	Betaine aldehyde dehydrogenase	+	+	+	+	+	+	+	+	+	+	+	+	+	+	+	+	+	+	+
dmt	permease	+	+	+	+	+	+	+	+	+	+	+	+	+	+	+	+	+	+	+
cbiF	precorrin-4/cobalt-precorrin-4 C11-methyltransferase	+	+	+	+	+	+	+	+	+	+	+	+	+	+	+	+	+	+	+
cbiQ	Cobalt transport protein	+	+	+	+	+	+	+	+	+	+	+	+	+	+	+	+	+	+	+
crtP	Phytoene dehydrogenase	+	+	+	+	+	+	+	+	+	+	+	+	+	+	+	+	+	+	+
cysC	Adenylyl-sulfate kinase	+	+	+	+	+	+	+	+	+	+	+	+	+	+	+	+	+	+	+
cytM	C-type cytochrome	+	+	+	+	+	+	+	+	+	+	+	+	+	+	+	+	+	+	+
dapA	4-hydroxy-tetrahydrodipicolinate synthase	+	+	+	+	+	+	+	+	+	+	+	+	+	+	+	+	+	+	+
deaD	ATP-dependent RNA helicase	+	+	+	+	+	+	+	+	+	+	+	+	+	+	+	+	+	+	+
desA	Delta(12)-fatty-acid desaturase	+	+	+	+	+	+	+	+	+	+	+	+	+	+	+	+	+	+	+
desD	Putative desferrioxamine (Siderophore) biosynthesis protein	+	+	+	+	+	+	+	+	+	+	+	+	+	+	+	+	+	+	+
folE	GTP cyclohydrolase-1	+	+	+	+	+	+	+	+	+	+	+	+	+	+	+	+	+	+	+
fus	elongation factor G (EF-G)	+	+	+	+	+	+	+	+	+	+	+	+	+	+	+	+	+	+	+
galU	UTP--glucose-1-phosphate uridylyltransferase	+	+	+	+	+	+	+	+	+	+	+	+	+	+	+	+	+	+	+

ggpP	Glucosylglycerate-phosphate phosphatases\haloacid dehalogenase	+	+	+	+	+	+	+	+	+	+	+	+	+	+	+	+	+	+	+
gpS	fullglucosyl-3-phosphoglycerate synthase\hypothetical protein	+	+	+	+	+	+	+	+	+	+	+	+	+	+	+	+	+	+	+
ggtB	Gammaglutamyltranspeptidase	+	+	+	+	+	+	+	+	+	+	+	+	+	+	+	+	+	+	+
ggtD	putative ABCtype sugar transport system, permease component	+	+	+	+	+	+	+	+	+	+	+	+	+	+	+	+	+	+	+
glgA	Glycogen synthase	+	+	+	+	+	+	+	+	-	+	+	+	+	+	+	+	+	+	+
glgB	1,4-alpha-D-glucan:1,4-alpha-D-glucan 6-glucosyl-transferase	+	+	+	+	+	+	+	+	+	+	+	+	+	+	+	+	+	+	+
glgC	glucose-1-phosphate adenylyltransferase	-	+	+	+	+	+	+	+	+	+	+	+	+	+	+	+	+	+	+
glgP	glycogen phosphorylase	-	+	+	+	+	+	+	+	+	+	+	+	+	+	+	+	+	+	+
gpgP	Glucosylglycerate-phosphate phosphatases\haloacid dehalogenase	+	+	+	+	+	+	+	+	+	+	+	+	+	+	+	+	+	+	+
gpgS	fullglucosyl-3-phosphoglycerate synthase\hypothetical protein	+	+	+	+	+	+	+	+	+	+	+	+	+	+	+	+	+	+	+
groEL1	60 kDa chaperonin 1	+	+	+	+	+	+	+	+	+	+	+	+	+	+	+	+	+	+	+
groEL2	60 kDa chaperonin 2	+	+	+	+	+	+	+	+	+	+	+	+	+	+	+	+	+	+	+
groES	10 kDa chaperonin	+	+	+	+	+	+	+	+	+	+	+	+	+	+	+	+	+	+	+
gshB	Glutathione synthetase	+	+	+	+	+	+	+	+	+	+	+	+	+	+	+	+	+	+	+
hflX	GTPase HflX	-	+	+	+	+	+	+	+	+	+	+	+	+	+	+	+	+	+	+
infA	Translation initiation factor IF-1	+	+	+	+	+	+	+	+	+	+	+	+	+	+	+	+	+	+	+
infB	Translation initiation factor IF-2	-	+	+	+	+	+	+	+	+	+	+	+	+	+	+	+	+	+	+
infC	Translation initiation factor IF-3	+	+	+	+	+	+	+	+	+	+	+	+	+	+	+	+	+	+	+
kdpA	potassium-transporting ATPase subunit A	+	+	+	+	+	+	+	+	+	+	+	+	+	+	+	+	+	+	+
kdpB	potassium-transporting ATPase B chain	+	+	+	+	+	+	+	+	+	+	+	+	+	+	+	+	+	+	+
kdpC	Potassium-transporting ATPase C chain	+	+	+	+	+	+	+	+	+	+	+	+	+	+	+	+	+	+	+
kdpD	sensor histidine kinase\potassium-transporting ATPase subunit D	+	+	+	+	+	+	+	+	+	+	+	+	+	+	+	+	+	+	+
kdpE	two component transcriptional regulator, winged helix family	+	+	+	+	+	+	+	+	+	+	+	+	+	+	+	+	+	+	+
ktrA	Na-activated K transporter subunit KtrA	+	+	+	+	+	+	+	+	+	+	+	+	+	+	+	+	+	+	+
moaA	Molybdenum cofactor biosynthesis protein A	-	+	+	+	+	+	+	+	+	+	+	+	+	+	+	+	+	+	+
mrp	multiple drug resistance protein	+	+	+	+	+	+	+	+	+	+	+	+	+	+	+	+	+	+	+

mscS	putative smallconductance mechanosensitive ion channel	+	+	+	+	+	+	+	+	+	+	+	+	+	+	+	+	+	+	+
mutS	DNA mismatch repair protein	+	+	+	+	+	+	+	+	+	+	+	+	+	+	+	+	+	+	+
narB	Nitrate reductase	+	+	+	+	+	+	+	+	+	+	+	+	+	+	+	+	+	+	+
ndhD2	NADH dehydrogenase subunit 4.	+	+	+	+	+	+	+	+	+	+	+	+	+	+	+	+	+	+	+
nusA	transcription termination/antitermination protein	+	+	+	+	+	+	+	+	+	+	+	+	+	+	+	+	+	+	+
nusG	transcription termination/antitermination protein	+	+	+	+	+	+	+	+	+	+	+	+	+	+	+	+	+	+	+
pbp	penicillin-binding protein.	+	+	+	+	+	+	+	+	+	+	+	+	+	+	+	+	+	+	+
pgm	phosphoglycerate mutase	+	+	+	+	+	+	+	+	+	+	+	+	+	+	+	+	+	+	+
pth	peptidyl-tRNA hydrolase	+	+	+	+	+	+	+	+	+	+	+	+	+	+	+	+	+	+	+
rbpA	RNA polymerase-binding protein	+	+	+	+	+	+	+	+	+	+	+	+	+	+	+	+	+	+	+
rlpA	endolytic peptidoglycan transglycosylase	+	+	+	+	+	+	+	+	+	+	+	+	+	+	+	+	+	+	+
rlpR	amino acid adenylation protein	+	+	+	+	+	+	+	+	+	+	+	+	+	+	+	+	+	+	+
rv3030	S-adenosylmethionine-dependent methyltransferase	-	+	+	+	+	+	+	+	+	+	+	+	+	+	+	+	+	+	+
*rv*3031	putative branching enzyme gene	+	+	+	+	+	+	+	+	+	+	+	+	+	+	+	+	+	+	+
*rv*3032	glycogen synthase	+	+	+	+	+	+	+	+	+	+	+	+	+	+	+	+	+	+	+
tigF	RNA polymerase sigma factor SigF	-	+	+	+	+	+	+	+	+	+	+	+	+	+	+	+	+	+	+
tus	sucrose synthases	+	+	+	+	+	+	+	+	+	+	+	+	+	+	+	+	+	+	+
tgt	tRNA-guanine transglycosylase	+	+	+	+	+	+	+	+	+	+	+	+	+	+	+	+	+	+	+
tig	protein homologous to trigger factor (TF)	+	+	+	+	+	+	+	+	+	+	+	+	+	+	+	+	+	+	+
treC	putative trehalose synthase, TreS-like	+	+	+	+	+	+	+	+	+	+	+	+	+	+	+	+	+	+	+
treT	putative glycosyltransferase, group 1	+	+	+	+	+	+	+	+	+	+	+	+	+	+	+	+	+	+	+
treX	glycogen debranching enzyme/alfa-amylase	+	+	+	+	+	+	+	+	+	+	+	+	+	+	+	+	+	+	+
treY	maltooligosyltrehalose synthases	+	+	+	+	+	+	+	+	+	+	+	+	+	+	+	+	+	+	+
treZ	maltooligosyltrehalose trehalohydrolases	+	+	+	+	+	+	+	+	+	+	+	+	+	+	+	+	+	+	+
tsf	elongation factor Ts	-	+	+	+	+	+	+	+	+	+	+	+	+	+	+	+	+	+	+

Genes selected according [94,95].
